# Local Relaxation Phenomena in Epoxy Resins in the Temperature Range from −150 °C to +150 °C

**DOI:** 10.3390/polym17243318

**Published:** 2025-12-16

**Authors:** Viktor A. Lomovskoy, Dmitry A. Trofimov, Svetlana A. Shatokhina, Nadezhda Yu. Lomovskaya, Igor D. Simonov-Emelyanov

**Affiliations:** 1 Frumkin Institute of Physical Chemistry and Electrochemistry, Russian Academy of Sciences (IPCE RAS), 119071 Moscow, Russia; lomovskoy49@gmail.com (V.A.L.); lomo335@yandex.ru (N.Y.L.); 2 JST “NPO Stekloplastik N.N. Trofimov”, 141551 Solnechnogorsk, Russia; d.trofimov@npostek.ru; 3 Lomonosov Institute of Fine Chemical Technologies, MIREA—Russian Technological University, 119571 Moscow, Russia; simonov@mitht.ru

**Keywords:** internal friction spectra, temperature–frequency dependences, local dissipative processes, relaxation time, shear modulus defect, epoxy oligomers

## Abstract

This study and theoretical analysis of local relaxation processes and their physicomechanical and physicochemical characteristics in uncured epoxy oligomers DER-330, ED-20, ED-16 and ED-8 were carried out in the dynamic mode of freely damped torsional oscillations excited in specimens of the investigated systems. Internal friction spectra and temperature dependences of the frequency of free damped oscillations were obtained within the temperature range covering both the solid and liquid states of the epoxy oligomers. Based on the phenomenological models of a standard linear solid and the Maxwell model, the energetic and relaxation characteristics for each local dissipative process, as well as the temperature changes in strength properties (considering the defects of the shear modulus of the relaxation process) of the system as a whole, were calculated.

## 1. Introduction

Epoxy oligomers differ in their properties from other polymeric materials and play an important role in the automotive, aerospace, shipbuilding, and other industries [1,2,3,4,5,6,7,8]. Their widespread use is due to the unique combination of performance characteristics of cured epoxy oligomers and the high processability of epoxy resins [9].

Epoxy polymers exhibit high static and impact strength, hardness, and wear resistance. They are characterized by significant thermostability and heat resistance. Many solid surfaces form strong adhesive bonds with epoxy polymers [10], which determines their application as compounds, adhesives, paints, varnishes, and coatings. The structure of epoxy oligomers—their molecular weight, chain distribution, and the presence of functional groups—has a substantial influence on the final properties of polymer systems, such as mechanical strength, thermal stability, adhesion, and relaxation behavior, all of which are directly related to the chemical structure of the resin [11,12].


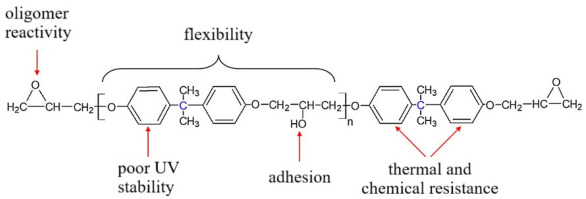


However, their behavior in the uncured state remains insufficiently studied, especially from the standpoint of molecular dynamics and relaxation processes. Understanding relaxation behavior is important for optimizing storage, transport, and processing conditions. Relaxation spectrometry methods—specifically, internal friction spectra and temperature–frequency dependences in the regime of free damped torsional oscillations—allow one to investigate molecular mechanisms of chain and bond motion, as well as to reveal features of structural relaxations from in terms of the atomic–molecular theory of matter of the studied systems.

A systematic analysis of epoxy resins and their constituents is thus necessary for a comprehensive understanding of polymer structure, dynamics, and properties, providing a scientific basis for developing advanced materials with tailored performance characteristics [13].

The aim of this study is to investigate the relaxation behavior of uncured epoxy oligomers by means of internal friction spectra and temperature–frequency dependences using relaxation spectrometry under freely damped torsional oscillations, to identify molecular mobility mechanisms and structural relaxations in these materials, and to perform their theoretical analysis.

### Relaxation Properties

Before addressing the relaxation properties of epoxy polymers, let us recall the general concepts of relaxation behavior and relaxation spectra [14,15,16,17]. Relaxation properties—that is, the ability of a material disturbed from equilibrium to return to it—are determined by the sum of all types of molecular motion: rotational, vibrational, and translational. By decomposing this multitude of motions into a set of harmonic oscillations, one arrives at a spectral characterization of matter. Formally, the kinetics of the process of change of the relaxation modulus $g\left(t\right)$ can be expressed as:

(1) gt=∑i=1nhie−tτi,

where $g\left(t\right)$ is the kinetic modulus, t is the running time, $h_{i}$ is the relaxation spectrum, and $\tau_{i}$ is the relaxation time.

In this case, the entire process is expressed as the sum of n independent elementary relaxational transitions. In the continuous case

(2) gt=∫τ=0∞hτe−tτdτ.

The relaxation spectrum $h_{i}$ or $h\left(\tau \right)$ as a function of relaxation time τ (or $\tau_{i}$) may also be represented as $H\left(\tau \right)=\tau \cdot h\left(\tau \right)$, allowing the wide range of relaxation times to be expressed on a compact logarithmic scale:

gt=∫τ=0∞Hτe−tτdlnτ.

The frequency dependence of the relaxational process is the Fourier transform of expression (2), namely:

(3) $$G*\left(\omega \right)=i\omega \int_{0}^{\infty }g\left(t\right)e^{-i\omega t}dt,$$

where ω is the frequency, $G*\left(\omega \right)$–complex modulus of elasticity.

As can be seen, there is a one-to-one correspondence between $G*\left(\omega \right)$ and $g\left(t\right)$, i.e., knowing one quantity allows the other to be obtained. Since expression (2) is, in turn, the integral Laplace transform of the spectrum $h\left(\tau \right)$, the same correspondence exists in this case as well. The complexity arises because the experimentally measured quantities $G*\left(\omega \right)$ and $g\left(t\right)$ are typically obtained with errors, either random (noise) or systematic, and moreover within a limited range of time and frequency. The integral operator on the right side of Equation (2) is of a smoothing operator, meaning that intense perturbations of the function $h\left(\tau \right)$ appear significantly attenuated in the function $g\left(t\right)$. However, this means that minor perturbations (errors) of the experimental data can introduce significant uncertainty into the solution of the problem of finding the relaxation spectrum of $h\left(\tau \right)$. Cutting off the integration limits also serves as a source of distortion of the results of the inverse Laplace and Fourier transforms. The complex quantity $G*\left(\omega \right)$ is usually expressed in terms of its real and imaginary components:

(4) $$\begin{array}{l} G*\left(\omega \right)=G^{\prime }\left(\omega \right)+iG^{\prime \prime }\left(\omega \right),\\ G^{\prime }\left(\omega \right)=\int_{\tau =0}^{\infty }h\left(\tau \right)\frac{\omega^{2}\tau^{2}}{1+\omega^{2}\tau^{2}}d\tau ,\\ G^{\prime \prime }\left(\omega \right)=\int_{\tau =0}^{\infty }h\left(\tau \right)\frac{\omega \tau }{1+\omega^{2}\tau^{2}}d\tau . \end{array}$$

Their ratio, known as the **loss tangent** (mechanical, dielectric, etc.), is given by

(5) $$\tan\delta =\frac{G^{\prime \prime }\left(\omega \right)}{G^{\prime }\left(\omega \right)},$$

and is often used to identify individual relaxation transitions. The magnitude (intensity) of the peak on the internal friction spectra—$\tan\delta \left(T\right)$ or $\tan\delta \left(\omega \right)$—is typically associated with the concentration of the relaxing element (relaxator) [18]. To describe and classify local relaxation transitions, G. M. Bartenev introduced the concept of **relaxation spectrometry** [14,15]. Localization of relaxation processes means that each occurs (and can be treated) independently of the others. The parameters of each process—such as relaxation time, activation energy, etc.—are determined by the local structure of the polymer. This is the essence of relaxation spectroscopy as a method for studying polymer structure.

The existence of a broad spectrum of local relaxation times arises even in the simplest macromolecules, such as polyethylene [19,20,21,22,23,24,25], polyethylene oxide [26,27,28,29,30,31,32,33,34,35,36], and polyvinyl alcohol [37,38,39], where multiple relaxators can be distinguished. Vibrational–rotational motions of atoms, side groups, and crankshaft-type rotations of intra-chain atomic groups are all characterized by different relaxation times. An increase in molecular structural complexity naturally leads to changes in relaxation spectra and temperature–frequency dependences.

Under an external deforming action, the response of the investigated system depends on the total reaction of its structural–kinetic subsystems, which act quasi-independently within their own temperature–frequency ranges [40,41]. The reaction of each structural-kinetic subsystem manifests itself in its own temperature-frequency range, is quasi-independent of the reaction of other structural-kinetic subsystems and is determined by its physicomechanical and physicochemical characteristics.

This is because the elements of these subsystems—links, segments, amorphous, or crystalline phases [15,19,24,42,43,44]—possess their own structure. The transition of these elements from a thermodynamic and mechanical nonequilibrium state to equilibrium is determined by their **transition functions**, which are among the main factors causing temperature- and frequency-localized dissipative processes. These manifest on internal friction spectra as loss peaks of various intensities positioned at distinct temperature intervals [19,42,43,45,46,47,48,49,50,51,52,53]. The ensemble of these transitions has a decisive impact on the service performance of polymer products under diverse operating conditions. The dynamic characteristics of these transitions are evaluated via their transition functions, which describe the system’s response both to internal changes and to external perturbations. From variations in these parameters, valuable information about the internal structure of the studied systems can be inferred.

The existence of transitions from nonequilibrium to equilibrium states in a non-conservative system leads to thermodynamic irreversibility and, consequently, to dissipation (internal friction) of part of the externally applied mechanical energy. A quantitative measure of energy dissipation is the absorption coefficient Ψ, related to other dissipative characteristics as:

(6) $$\Psi =\frac{\Delta W}{2\pi W}=Q^{-1}=tg\delta =\frac{\lambda }{\pi },$$

where ΔW and W are the irreversibly dissipated and supplied energies, respectively; $Q^{-1}$ is the internal friction; δ is the phase shift angle between the external action and the system’s response; λ is the logarithmic decrement of the damping oscillatory process [16].

This relationship makes it possible to obtain experimental internal-friction spectra. Internal friction is defined as the irreversible dissipation within a material of a portion of the mechanical energy applied to deform it [16,54]. The internal-friction spectrum consists of local dissipative-loss peaks appearing at different temperature ranges.

The logarithmic decrement λ is defined as

(7) $$\lambda =\frac{1}{n}\ln\frac{\varphi_{\max}}{\varphi \left(t\right)},$$

where n is the number of oscillations between the oscillation with amplitude $\varphi \left(t\right)$ and the oscillation with amplitude $\varphi_{\max}$ in the temporal representation of the oscillatory process.

For each temperature T of the investigation, there will be a corresponding temporal representation of the oscillatory process (as given in Equation (7)) and its own value of the logarithmic decrement λ, as well as its own value of the frequency ν of the oscillatory process excited in the investigated sample.

Studies of epoxy-oligomer–hardener compositions have shown that the characteristics of the resulting systems are directly linked to the physicomechanical and physicochemical properties of both the epoxy oligomer and the hardener individually [2,55,56,57,58,59]. However, systematic investigations of these components over wide temperature ranges (−150 °C to +150 °C) and under dynamic mechanical loading have been scarce. Only scattered reference data on the properties and reactivity of epoxy hardeners and related polymers are found in the literature [1,60,61,62,63].

In the present work, for the first time, experimental internal-friction spectra $\lambda =f\left(T\right)$ and temperature dependences of oscillation frequency $\nu =f\left(T\right)$ were obtained in the regime of freely damped torsional oscillations excited in samples of epoxy oligomers over the temperature range −150 °C to +150 °C.

The study presents an analysis of physicomechanical (temperature dependence of shear modulus, shear-modulus defect for local dissipative processes, temperature domains of local inelasticity, and internal-friction mechanisms) and physicochemical characteristics (activation energy and relaxation time of each local dissipative process) and their variations for uncured epoxy oligomers across the temperature range encompassing their solid and liquid states. Theoretical analysis of the experimental results is based on phenomenological model representations of the **standard linear solid** and the **generalized Maxwell model** [42].

Experiments were carried out on epoxy oligomers that were in the solid state at the initial temperature (−150 °C) and transitioned to the liquid state during heating [15,64,65,66,67,68,69,70,71,72,73].

Mechanical, dielectric, and other spectra of polymers typically display several broad relaxation transitions. The most intense process at the highest temperature is traditionally termed the α-transition (primary relaxation), while those at lower temperatures are denoted β-, γ-, δ-, etc., and classified as secondary relaxations [18,74]. Many researchers associate them with different types of molecular motion in polymers. The most interesting, according to [75], is the systematization reflected in the works of V.I. Irzhak [18] and G.M. Bartenev [76]:

– a high-temperature *α*-transition, the maximum temperature of which correlates with the glass transition temperature of the polymer; usually the *α*-transition is associated with a cooperative movement of the segmental type [76,77];
– a low-temperature *β*-transition, the nature of which is still debatable for various polymers; however, according to most authors, the *β*-transition is caused by a smaller scale and cooperative movement (compared with the α–transition) of relatively small sections of the polymer chain [26,76,77,78]—for example, links or segments [74,79];
– the low-temperature *γ*-transition is associated with the smallest-scale processes among the listed transitions and, according to many authors, is caused by the movement of the smallest sections of the polymer chain (side group [76], aliphatic groups –CH_2_– in the main chain [80], etc.). It is observed at fairly low temperatures (below −100 °C) and rarely falls within the temperature range of studies. In addition, not all methods make it possible to visualize the *γ*-relaxation process (for example, by high-frequency experiments [81]). In dielectric spectra, *γ*-transitions are often not resolved and merge into a single blurred maxim [76].

According to the literature data, β-relaxation processes are associated with both the local movement of individual fragments of the polymer main chain (units or segments) [74,76,79] or side groups [74,79], and the presence of impurities (water, monomer, etc.) [37,79]. Nevertheless, most authors consider the movement of individual, relatively small sections of macromolecules to be the main cause of β-relaxation [26,78,82], for example, in epoxy meshes (crankshaft movement) [83,84,85].

The temperature dependence of relaxation times for β-transitions, as well as for γ-transitions, is described by the Arrhenius equation [76,86] with an activation energy of 30–70 kJ/mol and a preexponential coefficient of the order of 2 × 10^−13^ s [18]. The comparatively high activation barrier for β-relaxation processes (relative to γ) means that this relaxation can be associated with the movement of the entire molecule or conformational movements within it [87]. Some information about β-transitions in epoxy meshes is given in [75]. In most studies, the β-relaxation process in epoxy systems based on diane epoxy oligomers is associated with the movement of two main fragments:

– hydroxyester (glyceryl) group

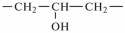
 [84,88,89,90], as the only common fragment of epoxy–amine meshes based on epoxy oligomers of various nature.

– fragment of diphenylolpropane (bisphenol A)

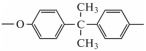
 [88,91,92], in particular, the movement of diester bonds in it [88], non-rotational bending movements of fragments containing benzene ring [88], the movement of aromatic rings themselves [85], etc. In the first case, it can be a crankshaft motion [84,88], the movement of a hydroxyester group bound by hydrogen bonds [88], etc. According to [83], the activation energy of the crank shaft motion is 46–63 kJ/mol, and for its implementation it is necessary to have a free volume where the “crank shaft” can rotate. According to [93], for epoxy–amine systems, β-relaxation of this fragment should be observed at about −93 °C; however, as can be seen from [75], these may be higher temperatures (up to −50 °C [92,94,95] and even higher).

It should be noted that in many works, experimental data and theoretical analysis are given for already cured epoxy systems. So, for example, in [89,96,97,98], the temperature position of the β-process is associated with the crosslinking density of a fully cured system using the example of various epoxy–amine meshes. In [99], a strong correlation was noted between the movement of molecular fragments and the amount of free volume in the polymer. With a relatively low crosslinking density, other effects are possible, such as a strong dipole–dipole interaction between the hydroxyester group and other polymer fragments (for example, terminal groups –CH_2_–CH_2_–CN in the hardener—a polypropylene imine dendrimer with terminal acrylonitrile groups [89]), which also reduces its mobility. According to [88], an increase in the concentration of phenolic groups in cured epoxy meshes does not affect their β-relaxation; therefore, many authors [100,101] do not take into account the contribution of the movement of benzene rings proper.

There are other explanations in the literature for the nature of the β-transition in epoxy meshes. For example, G.A. Pogany [102] believes that this transition may be associated with the movement of epoxy oligomer molecules attached to the grid with only one end, while A. Chatterjee and J. W. Gillespie, Jr. attribute it to the movement of amine–hardener fragments [95]. It is likely that these fragments can also contribute to the β-relaxation processes in epoxy meshes.

Sorbed water (for example, during the production of the material) has a significant effect on β-relaxation in epoxy–amine networks [75,92]. According to [90], the effect of sorbed water is significantly stronger than the effect of the molecular weight of the epoxy oligomer. Plasticization of meshes, including with water, reduces the intensity of the β-transition [90], which is realized not only in epoxy–amine meshes, but also in other systems [103,104,105].

In contrast to small-scale low-temperature transitions, the nature of the α-relaxation process in polymers is considered to be more or less established, and most authors associate it with the cooperative micro-Brownian motion of macromolecular chains [74,99,106,107], their large sections (segments) [108,109], the molecules themselves, or their ensembles [110]. It is generally assumed that the cooperative process of α-relaxation corresponds to vitrification [111], which is confirmed by experimental results [76]. In contrast to small-scale γ- and β-processes, the temperature dependence of the α-transition is usually not possible to express using the Arrhenius equation [18]. Nevertheless, it is believed that in a limited temperature range for it, *B* ≈ 5 × 10^−12^ s, *E_a_* = 50–70 kJ/mol [112]. Some experimental results [75], however, show that the activation energy of the α-relaxation for epoxy coatings and a number of linear polymers, determined by the dependence of the temperature of the maximum α-relaxation on the heating rate or frequency of the experiment, is often greatly overestimated [82].

Taking into account these features and the analysis of the literature sources, when considering relaxation processes in epoxy oligomers in detail, we divided them into γ- and β-processes, mainly based on the temperature values of the transition maxima: for the γ-transition—below −100 °C, and for the β-transition—from −100 °C to the α-relaxation.

In addition to the “basic” α-, β-, and γ-transitions, some authors identify “additional” relaxation processes for polymer systems, designated by them as λ-, μ-, δ-, and others, as well as the l-l transition [113]. The l-l transition observed during in many flexible polymers, at temperatures above the temperature of the α-process, $T_{\alpha }$ occupies a special place. According to [113], it is responsible for the transformation of a “liquid with a fixed structure” into a true liquid state and may be due to the presence of macromolecule associates, which in turn are nucleus of crystallization for polymers. Note that from this point of view, the l-l transition is unlikely for epoxy meshes due to a sufficiently high cross-link density and lack of tendency to crystallization; however, it is possible for the initial diane oligomers [114].

According to [114], this process for epoxy diane oligomers is associated with the transformation of a “liquid with a fixed structure” into a true liquid state; thus the $T_{ll}$ transition determines the physical boundary between the high-elasticity state and viscous-flow state of polymers. Their work investigated epoxy oligomers in an uncured state, grades: ED-22 (analog of the oligomer DER-330), ED-20, and ED-16. The temperatures of the l-l dissipative process obtained by the DSC method are as follows: 55.0 °C for ED-22; 57.8 °C for ED-20; 68.7 °C for ED-16.

In this paper, the main relaxation processes (γ-, β- and α-) occurring in uncured epoxy diane oligomers detected on the spectra of internal friction, and temperature–frequency dependences are considered.

## 2. Samples and Methods

### 2.1. Samples

The objects of the study were epoxy diane oligomers (EO) of the following grades: **DER-330** (Dow Chemicals, Midland, MI, USA), **ED-20**, **ED-16**, and **ED-8**.

These oligomers represent mixtures of oligomeric homologues with different fractional compositions and a general chemical formula [2,8,115,116,117]:


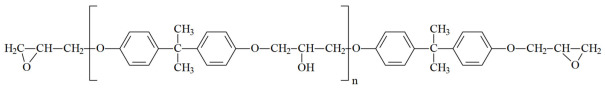
,

where *n* can range from 0 to 15 and beyond (up to ~200). As *n* increases, the viscosity of the oligomer also increases. The brand designations consist of the following: **E**—epoxy; **D**—diane; and numerical digits denoting the upper limit of the normative epoxy group content.

This selection of objects provides a wide range of molecular weights and viscosities typical of epoxy oligomers used in polymer composite manufacturing.

Table 1 presents passport data on the physicochemical and physicomechanical properties of the studied epoxy oligomers.

Industrial diane oligomers contain high-molecular-weight fractions that prevent crystallization. The contents of epoxy and hydroxyl groups determine the reactivity of the oligomer, while the distance between these groups (chain length) defines the cross-link density of the cured polymer [121].

Each oligomeric fraction contributes to the total viscosity of the epoxy resin. According to [122,123,124,125,126], the fractional composition of commercial epoxy oligomers (including those used in this study) can be described by the Flory equation:

(8) $$W(r)=r\cdot \lambda^{r-1}{\left(1-\lambda \right)}^{2},$$

with its help, it became possible to link the molecular weight distribution and fractional composition. In Equation (8), r is the polymerization degree, and λ is the degree of completion of the polycondensation reaction. Table 2 presents the fractional composition, the number of fractions contained in them, and their average molecular weights $M_{W}$, as well as the average weight average molecular weight of the studied oligomers calculated in [122].

The American-produced oligomer **DER-330** (Dow Chemical Company) is the analogue of the domestic ED-22. This oligomer is a colorless viscous liquid with a molecular mass of 350–400, epoxy content of 22.1–23.6%, and viscosity of 7–10 Pa·s at 20 °C.

The **ED-20** epoxy diane oligomer was among the first introduced to the Russian market. Despite new formulations, ED-20 remains widely used.

It is produced according to GOST 10587-84 and appears as a light-yellow viscous liquid with an epoxy content of 20–22.5%, molecular weight 390–430, and viscosity 20–40 Pa·s at 20 °C.

The next oligomer, **ED-16**, also produced under GOST 10587-84, closely resembles ED-20 but contains fewer epoxy groups and exhibits lower viscosity (3000–5000 Pa·s at 20 °C). Consequently, its gelation time is approximately twice shorter than that of ED-20 [1]. ED-16 is a light brown, poorly flowing liquid with a molecular weight of 450–650 and an epoxy content of 16–18%.

As expected, **ED-8** is even more viscous (300–350 Pa·s at 80 °C), has the lowest epoxy content (8–10%), and a higher molecular weight (1000–1400). It is a transparent solid and, like the previous oligomers, is manufactured according to GOST 10587-84.

**Figure 1:**
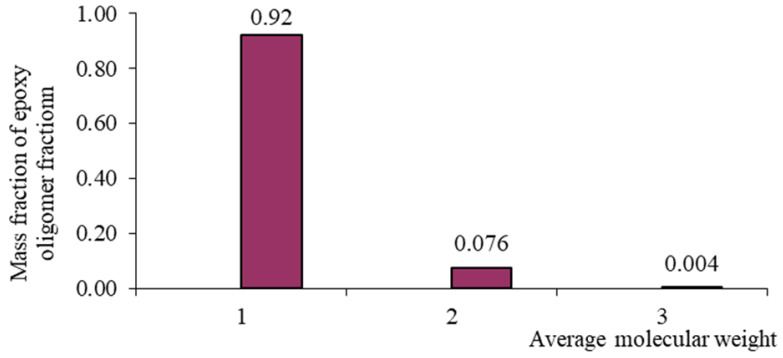
Mass–molecular distribution of the DER-330 oligomer.

**Figure 2:**
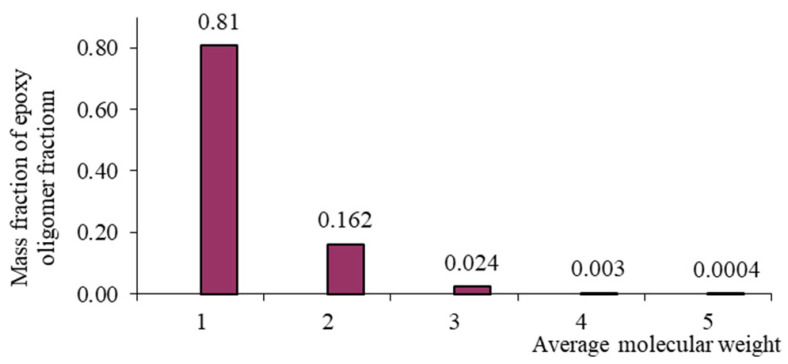
Mass–molecular distribution of the ED-20 oligomer.

**Figure 3:**
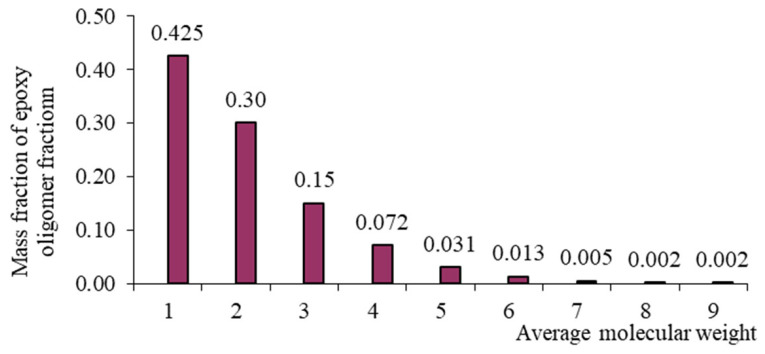
Mass–molecular distribution of the ED-16 oligomer.

**Figure 4:**
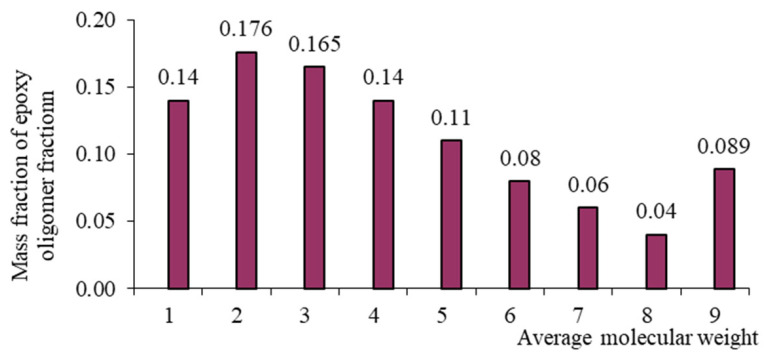
Mass–molecular distribution of the ED-8 oligomer.

From Table 1, it is evident that as the average molecular weight increases, the mass fraction of epoxy groups decreases (Figure 1, Figure 2, Figure 3 and Figure 4), the epoxy equivalent rises, and viscosity and fraction content both increase. Thus, the selected epoxy oligomers differ significantly in properties and are suitable for further investigation.

#### Sample Preparation

Experimental internal-friction spectra $\lambda =f\left(T\right)$ and temperature dependences of oscillation frequency $\nu =f\left(T\right)$ were obtained in the regime of freely damped torsional oscillations excited in specimens containing epoxy oligomers. Since epoxy oligomers are liquids, they cannot be directly incorporated into the oscillatory system. Therefore, a solid substrate was used, onto which the liquid oligomer was applied, forming a composite specimen of the type substrate–oligomer.

The substrate acts as the elastic component of the composite across the entire temperature range, while the oligomer responds elastically in its solid state ($T<T_{m}$) and viscoelastically in its liquid state ($T<T_{m}$).

Thus, within $-150\;{}^{\circ}C\leq T\leq T_{m}$, the sample behaves as a two-solid-phase composite (substrate + solid oligomer), and within $T_{m}\leq T\leq +150\;{}^{\circ}C$, it behaves as a solid–viscoelastic composite. In both cases, the samples were suitable for inclusion in the torsional-oscillation setup [127].

Special attention was given to the substrate material and geometry (thickness *h*, width *b*, and fixed length *l* = *const*) to minimize its moment of inertia while maintaining mechanical stability. Substrate dimensions for all experiments were 60 × 5 × 0.1 mm.

Because the focus was on dissipative phenomena in the oligomer subsystem rather than the entire composite, the substrate material had to be chemically inert toward the oligomer within −150 °C to +150 °C.

The substrate requirements were:

1. No significant dissipative losses within −150 °C to +150 °C that could obscure oligomer peaks;
2. Minimal moment of inertia I_s_ to avoid influence on the oscillation process;
3. No chemical interaction with the applied oligomer layer;
4. It is necessary to take into account the adhesive contact interactions between the surface of the substrate (matrix) and the composite oligomer.

Based on these criteria, three substrate types were considered: copper, cellulose, and stainless-steel mesh. Among them, cellulose was chosen for this work due to its favorable combination of mechanical and chemical properties [128,129,130].

Cellulose is a linear homopolysaccharide composed of anhydro-β-D-glucopyranose units linked by 1–4 glycosidic bonds (empirical formula (C_6_H_10_O_5_)_n_ or [C_6_H_7_O_2_(OH)_3_]_n_). Epoxy oligomers such as DER-330, ED-20, ED-16, and ED-8 can interact with cellulose materials (paper, cardboard, wood fibers) depending on temperature and curing conditions. Due to its porosity and surface hydroxyl groups, cellulose enables hydrogen bonding or polar adhesion with epoxy groups, improving wetting and interfacial contact. Without a curing agent, the interaction is primarily physical, but in the presence of amine or acid hardeners, chemical bonding through epoxy-ring opening may occur.

Copper substrates were excluded due to their chemical reactivity with epoxy oligomers, while stainless-steel substrates exhibited additional intense internal-friction peaks that complicated analysis. Cellulose also had the lowest stiffness among materials of equal moment of inertia, improving sensitivity for evaluating the shear moduli of liquid oligomers in composite samples.

### 2.2. Methods

To evaluate the molecular mobility of epoxy oligomers, the internal friction method was employed, based on the excitation of freely damped torsional oscillations in the studied samples. This method allows one to obtain experimental internal-friction spectra $\lambda =f\left(T\right)$ over a wide temperature range, as well as temperature dependences of oscillation frequency, $\nu =f\left(T\right)$, for the oscillatory process excited in the investigated systems. The resulting experimental data make it possible to conduct a theoretical analysis of the obtained results from the standpoint of the atomic–molecular discrete structure of the studied materials (systems).

**Figure 5:**
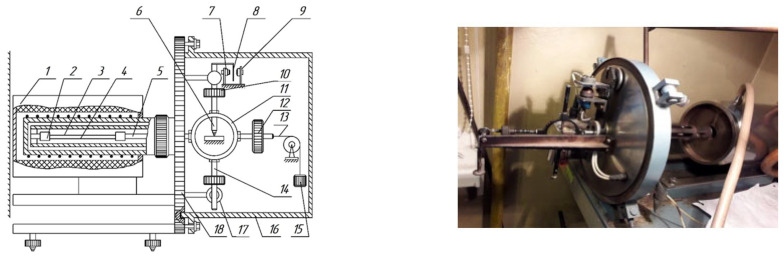
Horizontal torsional pendulum (block diagram). 1—Furnace housing; 2—fixed collet clamp; 3—investigated sample; 4—substrates used in the high-temperature range; 5—horizontal rod of the movable clamp; 6—support core of the oscillatory system; 7—lighting source; 8—optical shutter of the registration system; 9—photoelectric converter of the oscillation recording system; 10—inertia-adjustment weights; 11—central ring of the oscillatory system; 12—counterweight; 13—counterweight string; 14—pendulum beam; 15—weight for tension; 16—vacuum cover; 17—electromagnetic actuators for impulse excitation; 18—supporting vertical plate.

**Figure 6:**
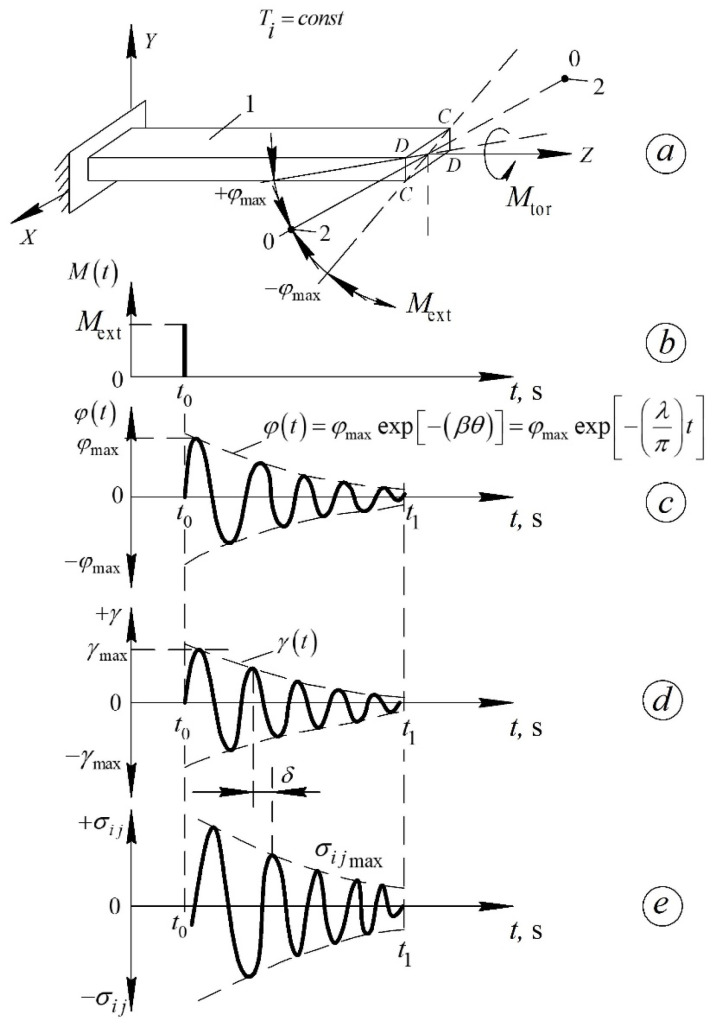
Epures of the freely decaying oscillatory process induced in the studied sample—(**a**) in the isothermal mode T=const by an impulse impact—(**b**) time dependence of the torsion angle φ(t)—(**c**) relative to the longitudinal axis Z of the sample. deformation arising in the sample—(**d**) and shear stresses $\sigma_{ij}$—(**e**) [40].

The polymer samples were fixed in the clamps of a horizontal torsional pendulum (Figure 5) and subjected to torsional deformation following the application of an external impulse torque $M_{ext}$ (Figure 6b) to the oscillatory system. The regime of external deformation is described by the relation:

$$M_{ext}=M\left(t\right)\cdot \delta (t)=\left\{\begin{array}{l} \;\;0\;\;\;\;\mathrm{by}\;\;t<t_{0}\\ M_{ext}\;\;by\;\;t=t_{0}\\ \;\;0\;\;\;\;\mathrm{by}\;\;t>t_{0} \end{array}\right.,$$

where δ(t) is the Dirac delta function.

The studied sample, being part of the oscillatory system, is twisted through an angle $\varphi_{0}$ (Figure 6c), corresponding to the initial amplitude of relative deformation $\gamma_{0}\approx 10^{-4}$. After the external impulse is applied, the sample performs damped torsional oscillations around the equilibrium position $\varphi \left(t\right)=0$ for a time interval $t<t_{0}<t_{1}$.

The time dependence of deformation in the sample is given by:

(9) $$\gamma \left(t\right)=\gamma_{0}\exp\left[-\left(\frac{\lambda }{\pi }\right)\right]t,$$

where λ is the logarithmic decrement of the oscillatory process excited in the sample, determined for each temperature (in an isothermal regime) from the relation (7).

This experimental technique thus provides temperature dependences of both the logarithmic decrement $\lambda =f\left(T\right)=f\left(\delta \right)$ and the oscillation frequency $\nu =f\left(T\right)$ of the free damped process excited in the sample.

For each temperature T, the investigated composite specimen (substrate–oligomer) was excited into free damped torsional oscillations corresponding to the amplitude of initial deformation ε_0_. The oscillatory process was recorded continuously during heating in the temperature range −150 °C to +150 °C, with a heating rate of 2 °C/min inside the thermocryochamber. The measurement error did not exceed ≈ 5% [40].

This method makes it possible to analyze relaxation transitions in polymers by observing changes in:

- the internal-friction spectrum $\lambda =f\left(T\right)$—which reflects energy dissipation due to molecular motion, and
- the oscillation frequency $\nu =f\left(T\right)$—which reflects the elastic stiffness and inertia of the system.

Increases in $\lambda =f\left(T\right)$ correspond to enhanced dissipative molecular processes (local relaxations, segmental mobility), while decreases in $\nu =f\left(T\right)$ indicate a reduction in the effective shear modulus due to softening or structural transitions.

The combination of these two dependences provides a comprehensive picture of local relaxation dynamics in polymer systems, allowing correlation of experimental peaks with specific molecular mechanisms such as γ-, β- and α-relaxations, and to quantify their activation energies and relaxation times using the Arrhenius relation and phenomenological models.

## 3. Results and Discussion

### 3.1. Materials

**Figure 7:**
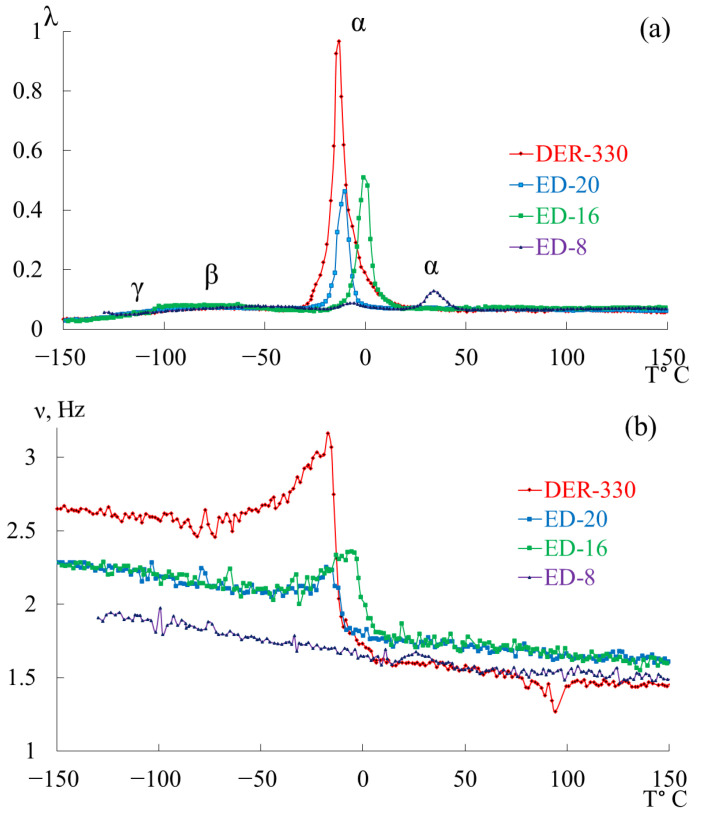
Internal friction spectrum $\lambda =f\left(T\right)$ (**a**); temperature dependence of frequency $\nu =f\left(T\right)$ (**b**) for epoxy oligomers: DER-330 (red line), ED-20 (blue line), ED-16 (green line), and ED-8 (violet line).

Figure 7 presents the internal-friction spectra $\lambda =f\left(T\right)$ (a) and the temperature dependences of oscillation frequency $\nu =f\left(T\right)$ (b) obtained from freely damped torsional oscillations in samples of epoxy oligomers deposited on cellulose substrates within the temperature range from −150 °C to +150 °C.

Upon initial inspection, the internal-friction spectra (Figure 7a) of DER-330, ED-20, and ED-16 show two main temperature regions where pronounced local dissipative processes occur:

- in the range from −120 °C to −40 °C, a low-intensity dissipative process ($\lambda_{i_{\max}}<0.1$, β−type relaxation) is observed;
- in the range from −30 °C to +20 °C, a high-intensity process ($0.4<\lambda_{i_{\max}}<1$, α-type relaxation) is detected.

The corresponding regions on the temperature–frequency curves (Figure 7b) exhibit characteristic variations in oscillation frequency. The highest frequencies at low temperatures are found for DER-330. ED-20 and ED-16 show very similar dependencies in both intensity and curve shape across the temperature range (except near the β-peak region). The ED-8 oligomer displays a notably different spectrum and $\nu =f\left(T\right)$ dependence, reflecting its unique molecular structure.

**Figure 8:**
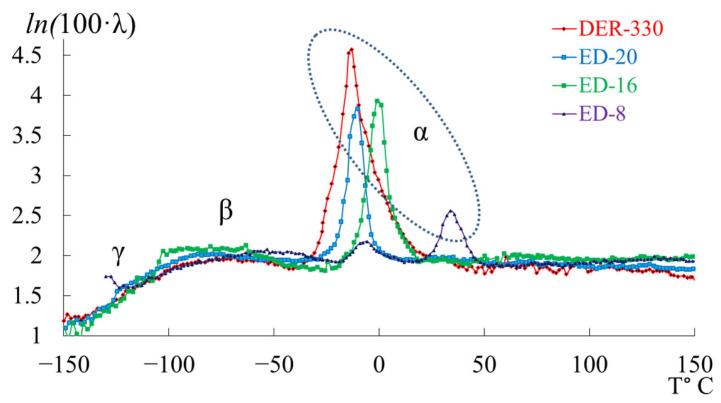
The logarithmic form of the internal-friction dependence $\ln\left(100\cdot \lambda \right)=f\left(T\right)$ for epoxy oligomers: DER-330 (red line), ED-20 (blue line), ED-16 (green line), and ED-8 (violet line).

In the graph (Figure 8), $\ln\left(100\cdot \lambda \right)$ is plotted along the ordinate axis, and the temperature in °C is plotted along the abscissa axis. The logarithmic dependence allows us to present a more distinct manifestation of small-scale dissipative processes (γ- and β-). High $\ln\left(100\cdot \lambda \right)$ values correspond to strong internal friction associated with intense molecular motion and relaxation transitions. Peaks indicate regions of maximum energy loss, corresponding to active structural rearrangements (γ-, β-, and α-relaxation transitions).

This logarithmic transformation helps to visualize the fine structure of dissipative processes, revealing the complex multicomponent nature of relaxation behavior in epoxy oligomers. Given the complexity of the loss peaks and associated “anomalies” on the temperature–frequency curves, each oligomer was analyzed separately and in detail.

### 3.2. DER-330

**Figure 9:**
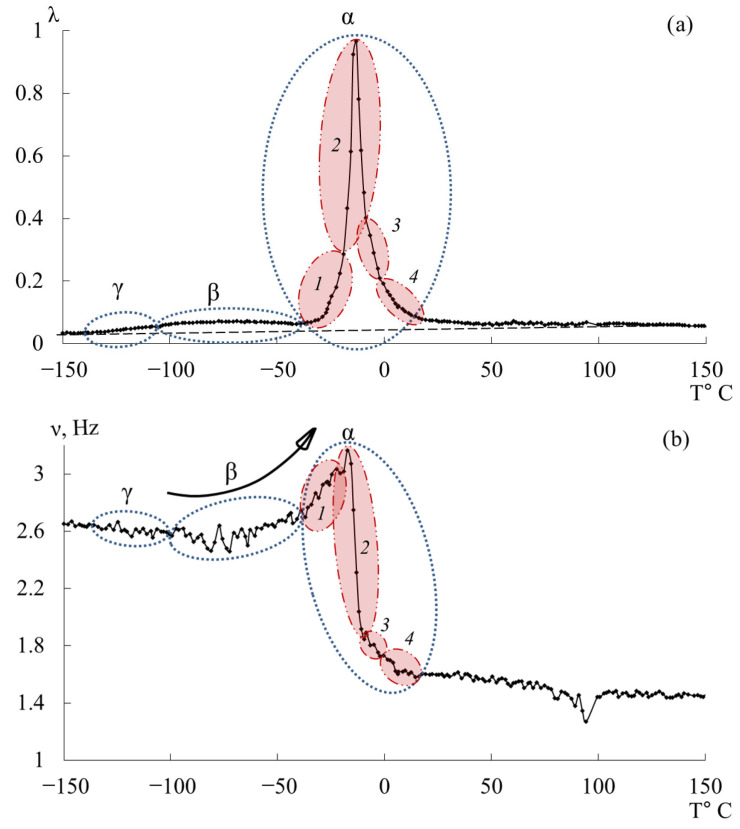
Internal friction spectrum $\lambda =f\left(T\right)$ (**a**); temperature dependence of frequency $\nu =f\left(T\right)$ (**b**) for epoxy oligomers DER-330.

A detailed examination revealed an additional low-intensity dissipative peak located between −130 °C and −110 °C, partially overlapped by the lower-temperature branch of the subsequent β-process. This process corresponds to the γ-relaxation, typically associated with the smallest-scale molecular motions (Figure 9a,b). Though weak, this γ-process is discernible in both $\lambda =f\left(T\right)$ and $\nu =f\left(T\right)$ curves through subtle variations in frequency. While the determination of precise activation and relaxation parameters is challenging due to the peak’s low intensity, approximate values can be estimated since the frequencies change only slightly with temperature.

At temperatures corresponding to the β-process, the oligomer remains in a glassy state, where polymer chains are largely immobilized and segmental mobility is frozen.

There is no unified view on the molecular fragments responsible for γ-relaxation; it may arise from the motion of caused by the movement of individual groups (side and in the main chain) or small chain segments. According to work [75], the most rational approach is that of V.I. Irzhak [18], according to which the γ-peak of dissipative losses is associated with the movement of molecular groups smaller than the size of the polymer segment (for example, side functional groups [113]). In an earlier work [131], where the internal friction spectra (obtained on horizontal and vertical torsion pendulums) exhibit a dependence similar to the experimental data of this work (for ED-22 resin—the analogue of DER-330), all small-scale relaxation processes are attributed to γ-transitions and associated with oscillatory-rotational movements of side groups (in this case, methyl (–CH_3_) and hydroxyl (–OH) groups).

The small temperature range (−150 °C to −130 °C) preceding this process is characterized by the virtually complete absence of relaxation processes both in the internal friction spectrum and in the temperature–frequency dependence (Figure 9a,b). Accordingly, it can be associated with the basic background level of elasticity, without relaxation movements. Previously, this range was not described in other works, including the work with similar spectra of epoxy diane polymer, where the temperature range included this section [131].

As temperature increases, a slight rise in the background of dissipative losses is observed. Such gradual background growth is also characteristic of other oligomers studied here. This background trend (dashed line in the figures) is not analyzed in detail since it lies beyond the scope of the current study.

The β-relaxation peak appears in the range −110 °C to −40 °C as a broad, low-intensity process encompassing multiple overlapping processes. The corresponding temperature–frequency curve exhibits a slight frequency increase within the same region (Figure 9b, black arrow), indicating that this β-loss peak has both relaxational and phase nature of internal friction. The methodology for determining the mechanisms of internal friction based on temperature-frequency dependences is described in detail in [41,132].

As already noted in the introduction, in many literary sources, experimental data and theoretical analysis are provided for already cured epoxy systems. However, in our case, the epoxy oligomers are in an uncured state; accordingly, the influence of crosslinking and its density on dissipative processes (including γ- and β-relaxation processes) on the presented experimental data is excluded. Accordingly, the presence (albeit weakly expressed) of the β-peak of dissipative losses in this case should be associated with the movement of individual, relatively small, sections of macromolecules. In favor of this assumption is the presence of a similar peak of dissipative losses on the internal friction spectra obtained, including by the authors of this work, for polymers of different structural structure, for example, LDPE and HDPE [19,25,44,78,133], PVA [38,39], PMMA [134] and POM [17,26]. Thus, β-relaxation processes are small-scale relaxation processes caused by vibrational–rotational movements of atoms and groups of atoms around the chain axis in the main polymer chain [17].

The complexity of the β-dissipative process, evident from the irregularities in the internal friction spectrum and the corresponding changes in the temperature–frequency dependence, can likely be attributed to the motions of more specific chain fragments, such as hydroxyether (glycerol) groups, bisphenol A fragments (diphenylolpropane), in particular the motions of diester linkages in them [88], non-rotational bending motions of fragments containing benzene rings [88], the motions of the aromatic rings themselves [85], and so on. However, this requires conducting a large number of additional studies of both epoxy oligomers and hardeners separately, as well as partially and fully cured systems of various structures.

Since the epoxy oligomer contains local physical nodes of the molecular network resulting from the formation of hydrogen and dipole–dipole bonds, relaxation transitions associated with the decay of these physical nodes should be expected. Such small-scale relaxation processes are caused by oscillatory-rotational forms of motion. In this case, the following types of interactions and related movements are possible: a hydrogen bond ($\mu_{H}$-relaxation) is formed by the interaction of a group –CH(OH)– of one chain with an oxygen atom of another chain; a dipole–dipole bond (π-relaxation) occurs between the polar oxygen atoms of neighboring chains. The breakdown of hydrogen at the $\mu_{H}$-transition temperature leads to a transition from one form of vibration (C–OH…O-vibration of one group –OH in the presence of a hydrogen bond) to another form (vibrations of the group –OH in the absence of a hydrogen bond), and rotation of the group –OH occurs [131].

Only a fraction of the potential physical nodes actually form due to the statistical distribution of reactive groups in space (since only those atoms or groups that happen to be close to each other can form bonds, such as hydrogen bonding or dipole–dipole interactions). As a result, a portion of side groups, such as –OH, remains free and undergoes vibrational–rotational motions. Only at higher temperatures, after the $\mu_{H}$-transition, do the previously bound –OH groups become free [131].

The third peak of dissipative losses is α, located in the temperature range from −40 °C to +20 °C, and is the most intense dissipative process on the spectrum of internal friction (Figure 9a). The temperature of the maximum of the α-peak of dissipative losses corresponds to the glass transition temperature of the un-crosslinked epoxy oligomer ED-22, which is essentially analogous to DER-330, and is −13 °C on the internal friction spectrum and −12.8 °C for ED-22 measured by DSC [114].

This α-peak is narrow and of high intensity but exhibits internal substructure, indicating the presence of several overlapping processes. In the same temperature region, the frequency curve (Figure 9b) shows an initial rise followed by a sharp drop, reflecting complex molecular dynamics (regions 1–4). Four subpeaks (1–4) within the α-process were thus identified (as a first approximation) by correlating $\lambda =f\left(T\right)$ and $\nu =f\left(T\right)$ (red ovals in Figure 9a,b).

Comparing Figure 9 with similar spectra obtained on a similar measuring pendulum [131], we see that in the region of the α-peak of dissipative losses, several processes are also distinguished: $\beta_{6}$, $\alpha_{1}$, α, $\alpha_{2}$, $\lambda_{1}$. These processes actually correspond to processes 1–4 presented on Figure 9a. Note that in work [131], this division is conducted only on the internal friction spectra, whereas in our presented work, the division was conducted taking into account the slightest changes on the internal friction spectra and simultaneously on the temperature–frequency dependencies, which are interconnected with each other.

As we can see from the internal friction spectra presented in work [131], the beginning of the α-process is preceded by $\beta_{5,6}$dissipative processes. In the spectra presented in our work, this area corresponds to the 1-subpeak of dissipative losses. At the same time, on the temperature–frequency curve, a dependence similar to the β-process is observed (an increase in frequency values, characterized only by some change in the rate of increase of these values). This is probably related to the fact that in this area, mobility related specifically to the β-relaxation process can indeed develop, but this does not exclude the beginning of the development of segmental mobility related to the α-process. In connection with this, we consider this question open and requiring more detailed consideration.

In work [131], the authors associate the α-relaxation process itself with the regional mobility of a microsection of the network, since the concept of a linear segment in a densely cross-linked polymer loses its meaning, and the kinetic unit of the α-relaxation process should be not a linear but a volumetric element, which includes a group of network chains (volumetric “segment”) [131]. It is obvious that in partially and fully cured epoxy–amine networks, the main relaxing unit cannot be individual macromolecules. The most probable relaxing entities are microvolumes of the network consisting of fragments of the initial monomers (EO and hardener), as well as sections formed due to the opening of epoxy rings during curing [75].

In our work, uncured oligomers are presented; accordingly, the influence of crosslinking on the α-relaxation process in Figure 9 is excluded. In this case, the α-peak of dissipative losses is associated with the unfreezing of the mobility of macromolecule segments and corresponds to the processes of mechanical and structural glass transition [76].

**Figure 10:**
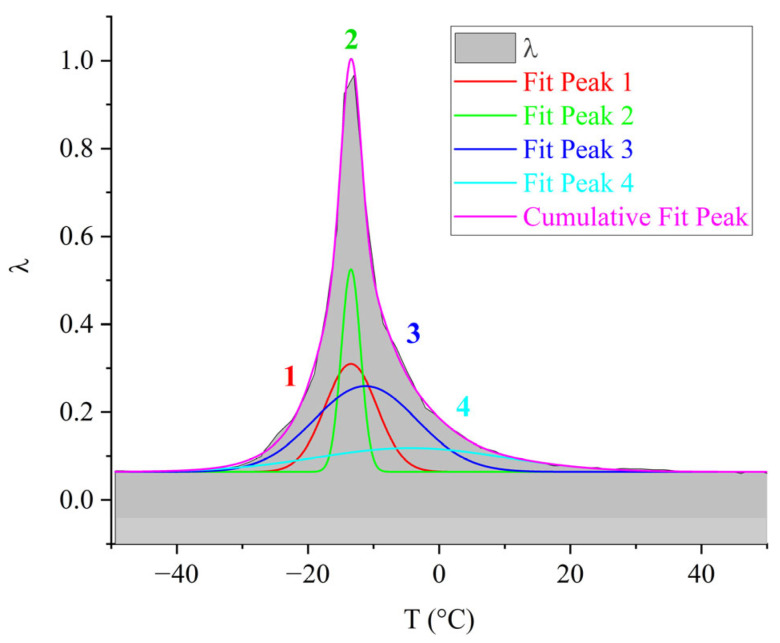
Decomposition of the α-relaxation loss peak using Gaussian fitting for the DER-330 oligomer.

For the purpose of visual representation of the contribution of each of the 1–4 subpeaks to the overall α-dissipative process, mathematical processing of the internal friction spectrum was carried out using the normal Gaussian distribution in the OriginPro program (Figure 10).

As seen from Figure 10, the overall α-dissipative process in DER-330 consists of four subpeaks that together describe the composite relaxation behavior. Among them, subpeak *2* provides the largest contribution to the total α-process. Subpeaks *1* and *3* contribute with nearly equal intensity; however, subpeak *3* is broader and spans almost the entire temperature interval of the α-process, while subpeak *1* is narrower and more localized in temperature. Subpeak *4* is the weakest in amplitude but extends across the entire α-relaxation region, which suggests that the molecular mobility responsible for this subpeak begins at the onset of glass transition and persists throughout the process. The broader width of a dissipative peak implies a larger number of relaxing subsystems with a range of characteristic parameters. Conversely, the narrower and more intense subpeak *2* likely corresponds to the activation of segmental mobility within a relatively small fraction of subsystems requiring higher energy input.

**Figure 11:**
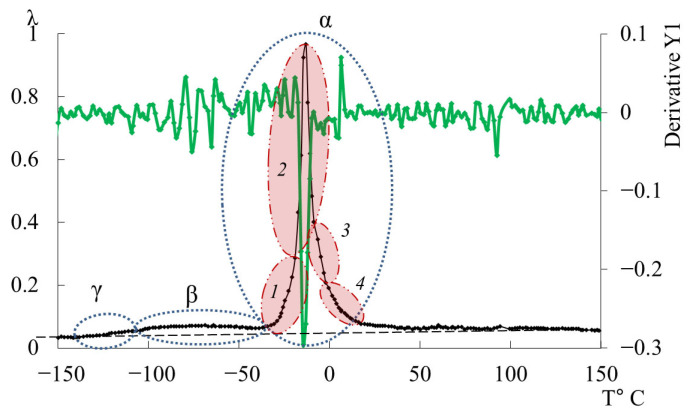
Internal-friction spectrum $\lambda =f\left(T\right)$ (main axis) and first derivative of the temperature–frequency dependence *dν*/*dT* (right-hand axis, green line) for the DER-330 oligomer.

In Figure 11, the primary ordinate axis shows the internal-friction spectrum, while the auxiliary right axis represents the first derivative of frequency with respect to temperature, *dν*/*dT*. This derivative characterizes the rate of change of oscillation frequency with temperature and reflects variations in stiffness, molecular mobility, and relaxation dynamics. In the region of the γ-transition (~−140 °C), *dν*/*dT* remains close to zero or varies only slightly, indicating a negligible temperature effect on frequency. The internal friction is low and nearly constant, signifying localized molecular motions that are only weakly temperature dependent.

In the β-transition region (~−70 °C), *dν*/*dT* begins to decrease (negative slope), corresponding to a reduction in frequency as temperature increases. This correlates with a rise in internal friction, indicating that developing mobility is increasing and dissipative energy losses are becoming significant. This suggests that the oscillatory process slows down as the temperature increases, and relaxation processes associated with the movement of chain sections increase inside the material.

In the region of the α-transition (~−10 °C), a sharp minimum in *dν*/*dT* is observed, demonstrating a sharp and significant change in frequency with temperature. At the same time, internal friction reaches a maximum, which reflects the main transition associated with vitrification. This sharp drop and subsequent recovery of the *dν*/*dT* values testify to complex dynamic processes: molecules transition from a glassy state to a more mobile one (abrupt increase in molecular mobility and decrease in stiffness), which significantly changes the properties of elasticity and internal friction.

Above the temperature of the α-process, in the temperature region ~90 °C, weak changes are noted on the first derivative curve, which are also noticeable on the temperature–frequency dependence in the form of some change in the slope angle of the curve (Figure 9b). Obviously, these processes are related to the l-l-dissipative process, previously detected by DSC in work [114], and may be due to the presence of macromolecular associates. The temperature ranges of the l-l-transition manifestation practically coincide with the experimental data of this work and the DSC curve from work [114].

### 3.3. ED-20

**Figure 12:**
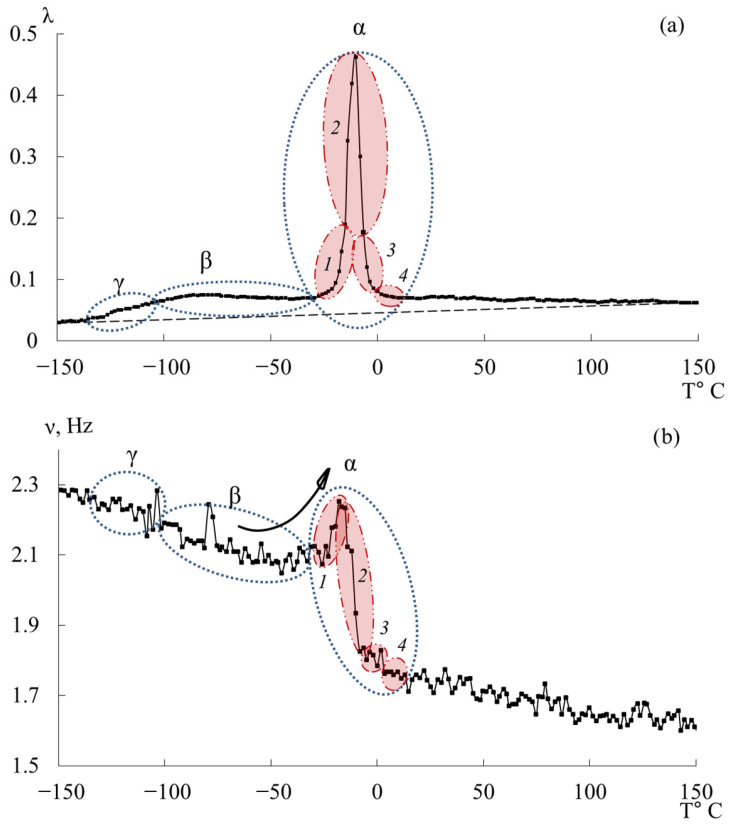
Internal friction spectrum $\lambda =f\left(T\right)$ (**a**); temperature dependence of frequency $\nu =f\left(T\right)$ (**b**) for epoxy oligomers ED-20.

Figure 12 presents the internal-friction spectrum $\lambda =f\left(T\right)$ (a) and the temperature–frequency dependence $\nu =f\left(T\right)$ (b) for the epoxy diane oligomer ED-20. The first dissipative losses peak, denoted γ, is similarly to the DER-330 oligomer, weakly expressed, absorbed by the low-temperature branch of the subsequent β-process, and located in the temperature range of −135 °C to −104 °C.

The small temperature interval preceding this process, similarly to the experimental data for DER-330, is characterized by the practical absence of relaxation processes both in the internal friction spectrum and in the temperature–frequency dependence (Figure 12a,b).

The second intense dissipative losses peak—β, located on the spectrum $\lambda =f\left(T\right)$ in the temperature interval from −104 °C to −30 °C, is also a low-intensity process. Similarly to the β-peak in the internal friction spectrum for DER-330, it is quite broad and includes a series of processes related to the mobilities of various subsystems. It is worth noting that the beginning of the active increase in frequency values in the region of manifestation of this dissipative losses peak, characteristic of DER-330, is observed at higher temperatures, above −50 °C.

The third dissipative losses peak—α, located on the spectrum $\lambda =f\left(T\right)$ in the temperature interval from −30 °C to +20 °C, is the highest-intensity dissipative process in the internal friction spectrum (Figure 12a). The peak is characterized by a narrow temperature interval of manifestation in the internal friction spectrum and includes a series of dissipative losses processes. There is a similar (to the DER-330 oligomer) temperature–frequency dependence structure and division into four subpeaks of the α-dissipative process in the internal friction spectrum, shown in Figure 12.

**Figure 13:**
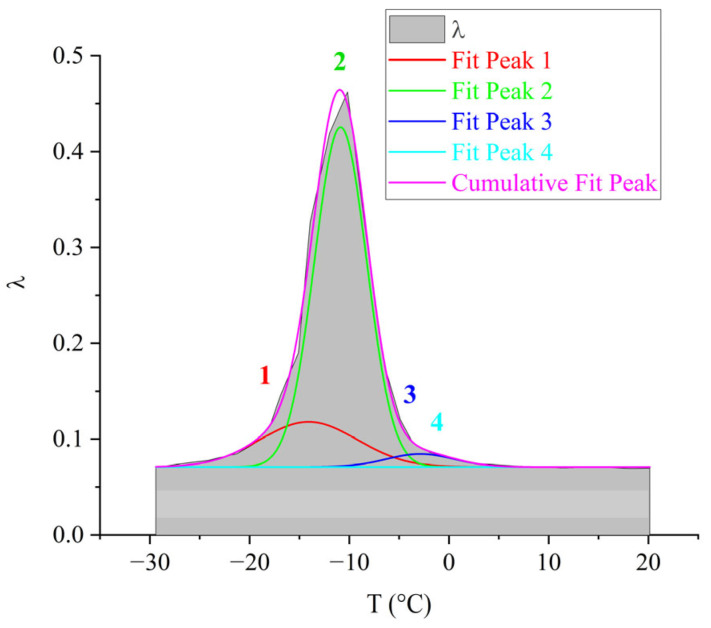
Decomposition of the α-relaxation loss peak using Gaussian fitting for the ED-20 oligomer.

From Figure 13, it can be seen that the greatest contribution to the overall α-dissipative process is provided by the *2*-relaxation subpeak. The next in intensity is the *1*-relaxation process, which contributes across almost the entire temperature range of the α-peak of dissipative losses. Peak *3* has low intensity and manifests in the highest temperature region of the overall α-relaxation peak. Process *4* degenerates. This is likely due to its very low intensity of damping of the oscillatory process, which causes it to be absorbed by the preceding subpeaks. Overall, both the intensity of the overall α-process and the contributions of each dissipative loss subpeak for the ED-20 oligomer show significant differences compared to the DER-330 oligomer. In this case, subpeak *2* provides a much larger contribution relative to the other subpeaks.

**Figure 14:**
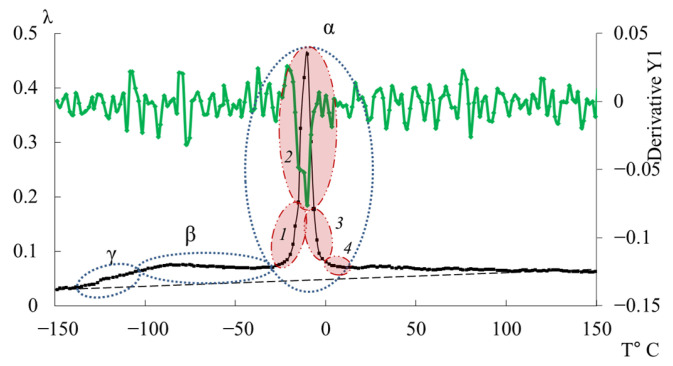
Internal-friction spectrum $\lambda =f\left(T\right)$ (main axis) and first derivative of the temperature–frequency dependence *dν*/*dT* (right-hand axis, green line) for the ED-20 oligomer.

From the analysis of the relationship between the internal friction spectrum (Figure 12a) and the first derivative (Figure 14), it was found that over the entire temperature range, the values of the first derivative are close to zero and exhibit weakly pronounced changes. The range of the first derivative values is smaller than that of the DER-330 oligomer. This indicates that the relaxation mobility is more uniform. The largest changes are characteristic of the regions where the highlighted dissipative processes (γ, β, α) manifest. At the same time, even for the α-process, the magnitude of the sharp peak has smaller (compared to DER-330) values.

### 3.4. ED-16

**Figure 15:**
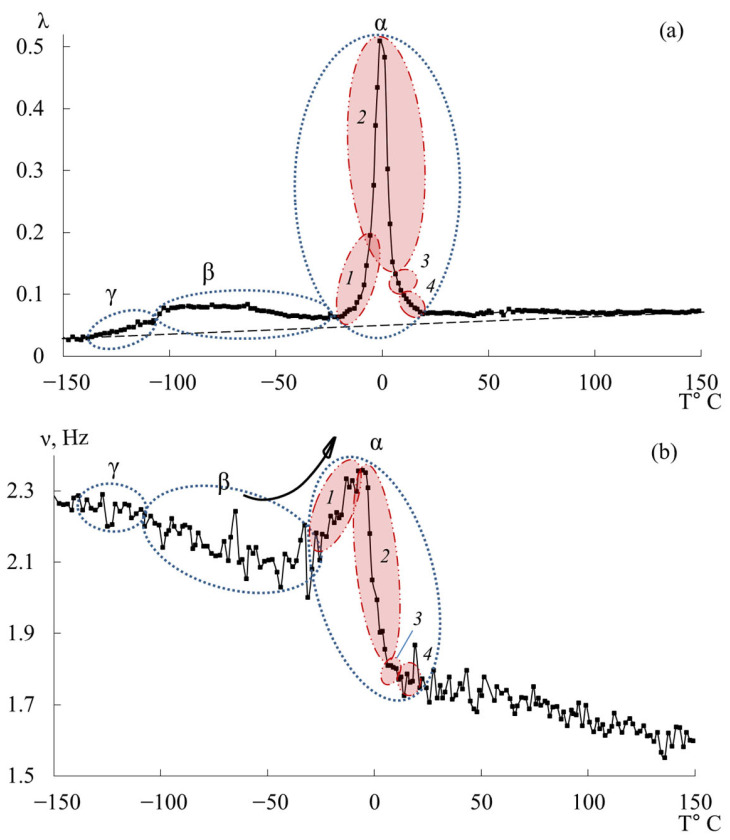
Internal friction spectrum $\lambda =f\left(T\right)$ (**a**); temperature dependence of frequency $\nu =f\left(T\right)$ (**b**) for epoxy oligomers ED-16.

Figure 15 shows the internal friction spectrum $\lambda =f\left(T\right)$ and temperature–frequency $\nu =f\left(T\right)$ dependence for the oligomer ED-16. The first peak of dissipative losses, denoted as γ, just like in the oligomers DER-330 and ED-20, is weakly expressed, absorbed by the low-temperature branch of the following β-process, and located in the temperature range from −138 °C to −108 °C. Compared to the experimental data of samples DER-330 and ED-20, in the ED-16 sample, this dissipative process is the most pronounced on the internal friction spectrum $\lambda =f\left(T\right)$.

The small temperature section preceding this process is also characterized by the practical absence of relaxation processes both on the internal friction spectrum and on the temperature–frequency dependence (Figure 15a,b).

The second intense peak of dissipative losses–β, located on the spectrum $\lambda =f\left(T\right)$ in the temperature range from −108 °C to −25 °C, is also a low-intensity process. Similar to the β-peaks on the internal friction spectrum for DER-330 and ED-20, it is quite wide and includes a series of processes related to the mobilities of various subsystems and is characterized by both relaxation and phase nature of the internal friction mechanism.

The third peak of dissipative losses–α, located on the spectrum $\lambda =f\left(T\right)$ in the temperature range from −25 °C to +20 °C, is the most high-intensity dissipative process on the internal friction spectrum, also characterized by a narrow temperature interval of manifestation on the internal friction spectrum (Figure 15a), and includes a series of dissipative loss processes. On the temperature–frequency dependence, there is an increase in frequency values, followed by a sharp decrease (Figure 15b).

**Figure 16:**
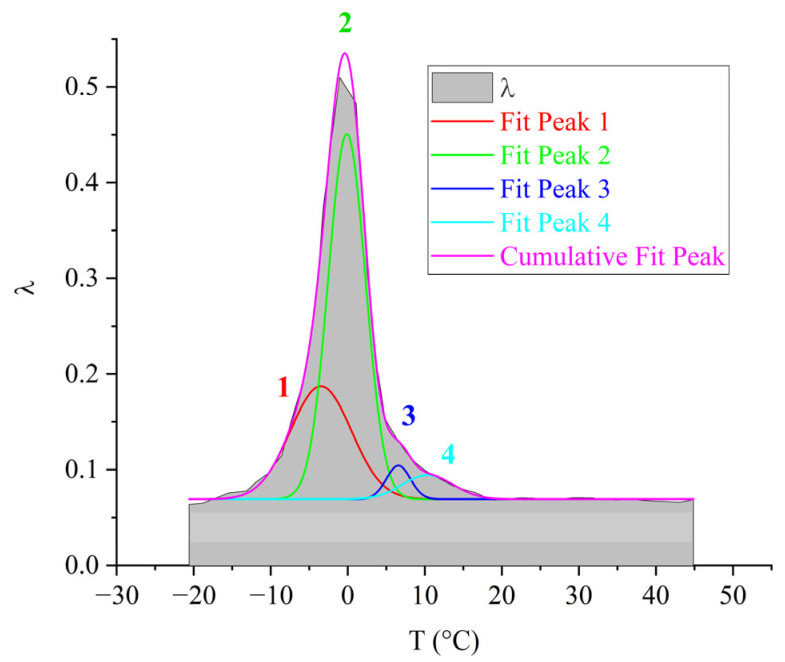
Decomposition of the α-relaxation loss peak using Gaussian fitting for the ED-16 oligomer.

The four subpeaks of dissipative losses, extracted using mathematical processing, entering into the α-relaxation process, are presented in Figure 16.

The largest contribution to the general α-dissipative process (Figure 16) is made by the *2*-relaxation subpeak (just like in the oligomers DER-330 and ED-20). The next in intensity—the *1*-relaxation process—contributes practically throughout the entire temperature range of the α-peak manifestation of dissipative losses. Peaks *3* and *4* have low intensity and manifest in the highest-temperature region of the general α-relaxation peak. They are slightly shifted along the temperature axis to the right, thereby changing the right branch of the general α-peak, making a visible contribution. Due to the fact that the range of their manifestation (according to Figure 16) is small, one can assume some locality of their manifestation.

**Figure 17:**
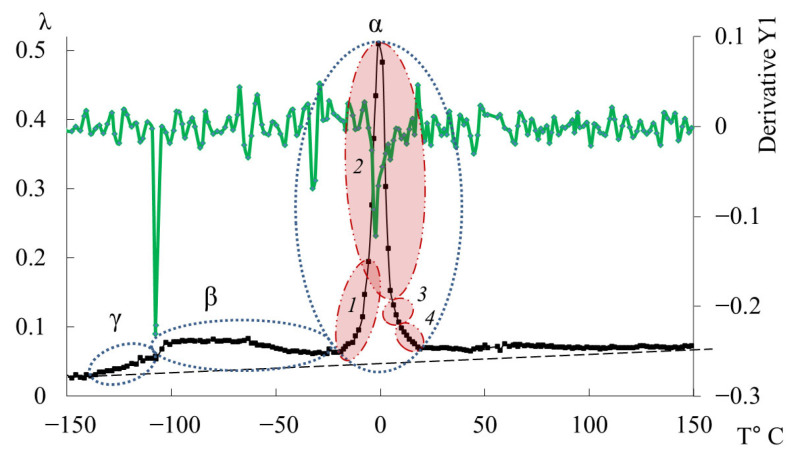
Internal-friction spectrum $\lambda =f\left(T\right)$ (main axis) and first derivative of the temperature–frequency dependence *dν*/*dT* (right-hand axis, green line) for the ED-16 oligomer.

Similar to the oligomer ED-20, the values of the first derivative *dν*/*dT* across the entire temperature range are close to zero and exhibit weakly expressed changes (Figure 17). The range of values of the first derivative is smaller than that of the oligomer DER-330 and more similar to the curve of the first derivative of the oligomer ED-20 (Figure 14). Accordingly, the relaxation mobility is more homogeneous (than in DER-330) and similar to that in ED-20.

### 3.5. ED-8

Upon a more detailed examination of the internal friction spectrum $\lambda =f\left(T\right)$ and the temperature–frequency curve $\nu =f\left(T\right)$ for the epoxy oligomer ED-8, a significant difference was found in the experimental dependencies compared to those for the previously described oligomers DER-330, ED-20, and ED-16. The intensities of all dissipative processes have much smaller values, and relative to the most high-intensity α-process, the β-processes do not have such a large difference in intensity.

**Figure 18:**
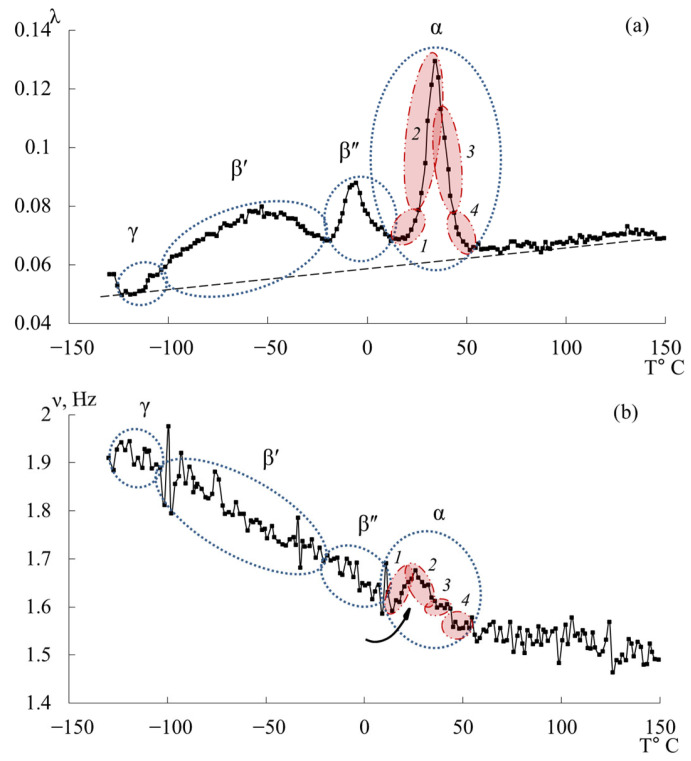
Internal friction spectrum $\lambda =f\left(T\right)$ (**a**); temperature dependence of frequency $\nu =f\left(T\right)$ (**b**) for epoxy oligomers ED-8.

The peak of dissipative losses, denoted as γ on the spectrum (Figure 18a,b), is located in the temperature range from −120 °C to −105 °C, is weakly expressed, and is partially absorbed by the low-temperature branch of the following β-process, similar to the previous oligomers.

The second intense peak of dissipative losses–β, is represented by two separate processes ($\beta^{\prime }$ and $\beta^{\prime \prime }$), located on the spectrum $\lambda =f\left(T\right)$ in the temperature intervals from −105 °C to −19 °C and from −19 °C to 16 °C. Both processes have a complex structure and consist of many outbursts, which is also noted on the temperature–frequency dependence (Figure 18b). The increase in frequency values in the manifestation area of these dissipative loss peaks (Figure 18b) indicates that this range is characterized by both relaxation and phase (locally) nature of the internal friction mechanism. Previously, similar experimental data for the oligomer ED-8 or other epoxy diane oligomers were not found in literary sources [76,77,100,113,131,135].

**Figure 19:**
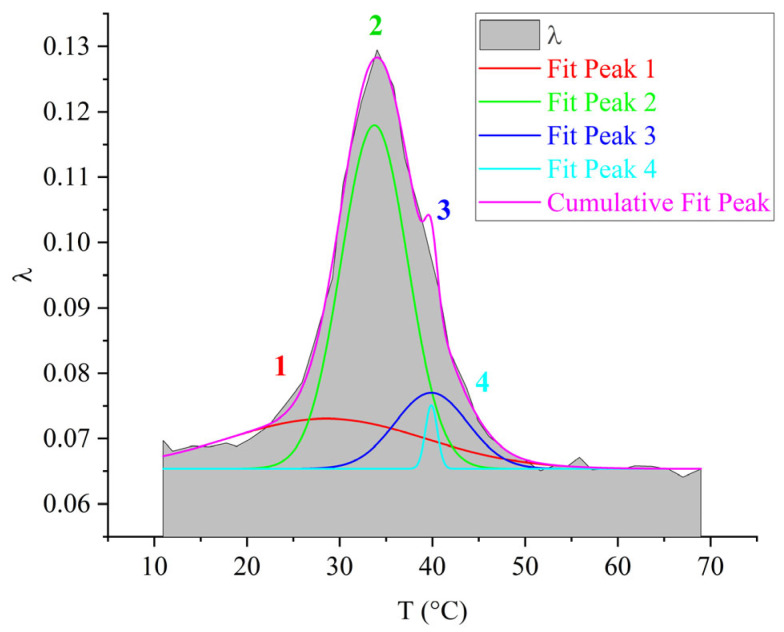
Decomposition of the α-relaxation loss peak using Gaussian fitting for the ED-8 oligomer.

The third peak of dissipative losses–α, is located on the spectrum $\lambda =f\left(T\right)$ in the temperature interval from 16 °C to 55 °C and is the most high-intensity dissipative process on the internal friction spectrum (Figure 18a). In addition to the highest intensity values, the peak is characterized by the narrowest temperature manifestation interval (Figure 18a) and has a complex structure. At the same time, a similar structure of the α-process on the internal friction spectrum (despite much smaller intensity values) and on the temperature–frequency dependence of the oligomer ED-8 with the oligomers DER-330, ED-20, and ED-16 is noted. By analogy with the above-described oligomers, this peak was decomposed into four subpeaks, marked on Figure 19 as areas *1*–*4* in red color. On the temperature–frequency dependence, an increase in frequency values is observed, followed by a sharp decrease (Figure 19, areas *1*–*4*).

The largest contribution to the overall α-dissipative process (Figure 19) is made by the *2*-subpeak of relaxation (as well as in the oligomers DER-330, ED-20, and ED-16). The *1*-relaxation process contributes throughout the entire temperature range of the α-peak of dissipative losses. Peaks *3* and *4* have low intensity and manifest in the highest-temperature region of the overall α-peak of relaxation. They are somewhat shifted along the temperature axis to the right; however, they are practically absorbed by the right branch of the overall α-peak of relaxation. Due to the fact that the range of their manifestation (according to Figure 19) is small, one can assume some locality of their manifestation.

**Figure 20:**
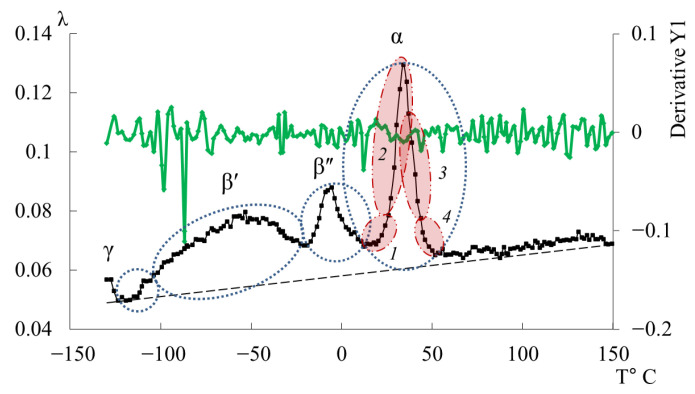
Internal-friction spectrum $\lambda =f\left(T\right)$ (main axis) and first derivative of the temperature–frequency dependence *dν*/*dT* (right-hand axis, green line) for the ED-8 oligomer.

Similarly to the ED-20 oligomer, the values of the first derivative over the entire temperature range exhibit weakly expressed changes (Figure 20). The range of the first derivative values is smaller than that of the DER-330 oligomer and differs from the first derivative of all previously presented oligomers (Figure 11, Figure 14 and Figure 17). Accordingly, the relaxation mobility is more homogeneous (than in DER-330) and similar to that in ED-20.

### 3.6. Calculation of Physicochemical and Physicomechanical Characteristics of Dissipative Processes

The calculation of physicomechanical and physicochemical characteristics for the γ-, β- and α-processes of dissipative losses was conducted based on the model representations of a standard linear solid. The solution to the differential equation of the standard linear solid in dynamic mode, taking into account the temperature–frequency dependence of the logarithmic decrement of the damped oscillatory process, is expressed as follows [19,40]:

(10) $$\lambda_{i}=2\lambda_{i_{\max}}\frac{\omega \tau }{1+{\left(\omega \tau \right)}^{2}},$$

where $\lambda_{i}$ and $\lambda_{i_{\max}}$ are the current and maximum values of the logarithmic coefficient of the damped oscillatory process for the i-th dissipative process; $\tau \equiv \tau_{i}=\frac{\eta }{G_{1}}$ is relaxation time of the i-th subsystem, causing the appearance of the dissipative loss peak on the spectrum $\lambda =f\left(T\right)$.

According to Deborah’s frequency–time relationship, $\lambda_{i}$ reaches its maximum at the peak of losses (when $\left(\lambda_{i}=\lambda_{i_{\max}}\right)$) under the condition specified by Equation (10).

(11) $$\omega \tau_{i}=1,$$

where the relaxation time $\tau_{i}$ corresponds to the relaxation time at the peak of dissipative losses in the spectrum $\lambda =f\left(T\right)$ and is defined by the Arrhenius equation:

(12) τ=τ0expERT,

where E is the activation energy of the dissipative process; $\tau_{0}\approx 1.6\cdot 10^{-13},\;s$ (for γ- and β-processes) and $\tau_{0}\approx 5\cdot 10^{-12},\;s$ (for α-process) are the theoretical value of the pre-exponential factor characterizing the oscillatory process of a relaxing particle at the bottom of the potential well for EO [18,131]; and R is the gas constant.

The frequency of the oscillatory process ν (determined experimentally from the dependence $\nu =f\left(T\right)$) is related to the angular frequency ω by the relationship ω=2πν. This allows for the determination of the relaxation time $\tau_{i}=\tau_{i_{\max}}$ at the peak of local dissipative losses $\lambda_{\max}$ based on the corresponding frequency value $\nu_{i}=\nu_{i_{\max}}$ and on the temperature dependence $\nu =f\left(T\right)$. For example, for the α-dissipative process observed in the internal friction spectrum for EO DER-330 (Table 3):

(13) $$\tau_{\alpha }=\frac{1}{2\cdot \pi \cdot 2.31}=0.069\;s\;.$$

The activation energy of these processes is determined from the Arrhenius dependence of the relaxation time τ on temperature (Equation (12)), taking into account Equation (13):

(14) Uαmax=RTαmaxlnταmaxτ0==8.314⋅260∗⋅ln0.0695⋅10−12=50469.83 Jmol≈50.5 kJmol.∗The temperature is given in K.

Calculations were carried out similarly for all processes of dissipative losses of the studied oligomers. All experimental and calculated data are presented in Table 3.

The activation energy values for the γ-relaxation process for the studied oligomers are on the order of 35 to 38 kJ/mol. It should be noted that for the temperature position of this dissipative process, its low intensity values on the internal friction spectra correspond to γ-processes; however, such high activation energy values (~36 kJ/mol) are characteristic, according to Izhak V.I.’s work [18], rather of β-relaxation. For γ-relaxation, the activation energy values are 10 to 20 kJ/mol [18], ~13 kJ/mol, corresponding to the unfreezing of rotation of the methyl group –CH_3_ [131]. However, alongside the mention of activation energy values in the literature, no information is provided on the exact temperature values and the corresponding frequency of the specific oscillatory process, which play a decisive role in the calculated activation energy values. Most often, only one thing is mentioned, which complicates the analysis of the presented characteristics. Accordingly, according to our calculations, such low activation energy values should correspond to lower temperatures of manifestation of the γ-relaxation process and much higher indicators of the oscillatory process frequency or much larger values of the pre-exponential coefficient, which is probably the reason.

It is also worth mentioning one of the most important quality indicators of epoxy resins—their reactivity. Reactivity can be expressed in several ways. In Russian-language scientific sources, the mass content of epoxy groups in the resin or composition is most often given in percent. However, a more convenient indicator for calculating the ratio of resin and hardener is the epoxy equivalent or epoxy equivalent weight (weight per epoxy equivalent (WPE)). This is the mass of the resin (usually in grams) containing 1 mole of epoxy groups [136]. There are also such characteristics as the number of epoxy groups (in grams or moles) in a certain amount of resin (usually in 100 g) [136]; epoxy value—how many mole equivalents of epoxy oxygen in 100 g of resin; and percentage content of epoxy ring oxygen in the resin [137,138].

Another way to describe the reactivity of the resin, which, however, is rarely standardized, is its functionality *f*, i.e., how many functional groups are in one molecule of the resin [137,138]. For the resins described, the information about functionality in terms of epoxy groups is most important. Epoxy resins consisting of molecules with terminal epoxide groups are considered bifunctional (*f* = 2); however, in production it is impossible to create an ideal product—the functionality of all molecules of the product is not always identical, which can lead to microstructural defects during curing. The distribution by functionality type allows predicting the defectiveness of the epoxy material structure. With increasing molecular weight, the content of mono- and non-functional molecules in the resin increases. In addition, there are polyfunctional resins. The described parameter in them varies over a wider range depending on the tasks set and the resin production technology [120].

The presence of microstructural defects in the structure and the distribution by functionality type will affect the internal friction spectra and temperature–frequency dependencies over the entire temperature range. We assume that the presence of mono- and non-functional molecules will lead to the complication of the oligomeric system structure, changes in the number of intermolecular interactions, and, consequently, an increase in the activation energy values in the region of the γ-relaxation process manifestation. Since, as is known, the presence of hydroxyl and terminal epoxy groups, as well as benzene nuclei, leads to strong intermolecular interactions in them (hydrogen bonds, π-π-interactions).

The value of the pre-exponential factor in the Arrhenius equation when calculating the activation energy also plays a key role. As noted above, in this work, the value $\tau_{0}\approx 1.6\cdot 10^{-13},\;s$ s is adopted for γ- and β-relaxation processes. At $\tau_{0}\approx 1\cdot 10^{-9},\;s$, the activation energies will take the following values: 23.9 kJ/mol for DER-330; 23.6 kJ/mol for ED-20; 23.7 kJ/mol for ED-16; and 25.4 kJ/mol for ED-8. These activation energy values are closer to 20 kJ/mol, but still higher, even despite the fact that the largest value of the pre-exponential factor was taken, according to work [18] ($\tau_{0}\approx 10^{-9}-10^{-12},\;s$).

According to work [18], for larger-scale β−processes, characteristic values are $\tau_{0}\approx 2\cdot 10^{-13},\;s$, and activation energy Uβ≈30−70 kJmol, which correlates with the calculated data obtained in our work. On the internal friction spectra and temperature–frequency dependencies for the oligomer samples presented in this work, it can be seen that the β-process is complex, including a series of processes of different natures (relaxational and non-relaxational). However, separating them into components and calculating all characteristics for them is difficult and does not guarantee an accurate result. Therefore, one value of all characteristics is given for the maximum intensity of this process. The activation energy values (Table 3) correspond to the literature data [18,131].

For α-relaxation processes, characteristic values are $\tau_{0}∼5\cdot 10^{-12},\;s$, and activation energy Uα≈50−70 kJmol [18], which also corresponds to the values obtained by us. Meanwhile, in comparison with work [131], there is a strong difference in the manifestation temperatures of the α-relaxation process and the activation energy values. This is due to the fact that the oligomer samples investigated in the cited work are cured. Accordingly, a shift of α-processes to higher temperature regions and expansion along the temperature axis of β-relaxation processes occurs during the curing of epoxy oligomers. The values of temperatures and activation energies in the loss peak for cured (according to work [131]) and uncured epoxy oligomer (studies presented in this work) for γ- and β-processes are practically similar and differ significantly for α-relaxation processes. It was not possible to estimate the change in relaxation times relative to the presence of the curing process due to the lack of data in the literature.

**Figure 21:**
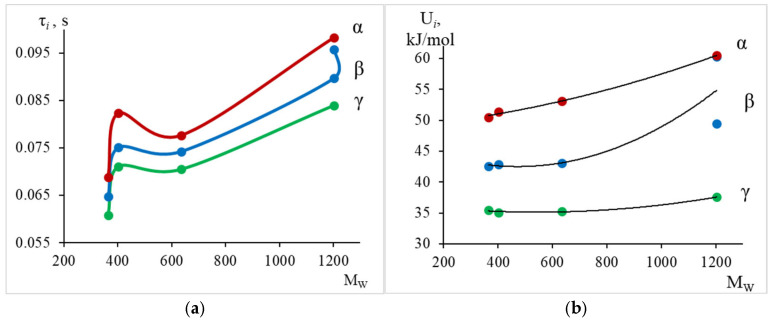
The dependence of relaxation time (**a**) and activation energy (**b**) for the γ- (green line), β- (blue line), α- (red line) dissipative relaxation processes on the average molecular weight.

In Figure 21, the change in relaxation times and activation energies for all three dissipative processes is graphically represented.

Thus, from the calculated data (Table 3) and Figure 21, we see that with increasing weight-average molecular mass, there is a slight increase in the values of relaxation time and activation energy for each dissipative process. We also note a certain difference in the behavior of the curve for the oligomer DER-330, which is an analogue of the Russian oligomer ED-22. The greatest changes are observed in the region of α-relaxation manifestation, where an increase in activation energy from 50.5 kJ/mol to 60.5 kJ/mol is observed.

**Figure 22:**
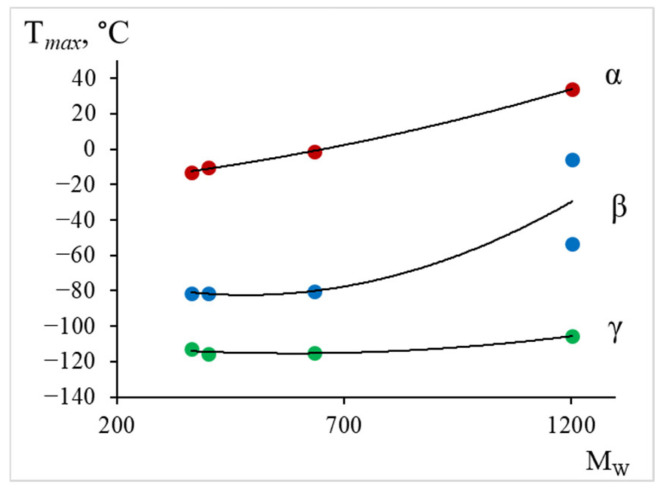
The dependence of the maximum temperature for the γ- (green line), β- (blue line), α- (red line) dissipative relaxation processes on the average molecular weight.

From the tabular data (Table 3) and Figure 22, we observe a similar dependence, namely: the largest changes in the temperature of the peak of dissipative losses are noted in the region where the α-relaxation process manifests.

The temperature dependence of the frequency $\nu =f\left(T\right)$ of the free damped oscillatory process excited in the studied system allows determining the temperature dependence of the shear modulus $G\left(T\right)$ of the material from which the studied sample is made, over the entire temperature range of the investigation. The experimental dependencies $\nu =f\left(T\right)$ (Figure 7b, Figure 9b, Figure 12b, Figure 15b and Figure 18b) show that in certain temperature intervals, where local dissipative processes are observed in the form of loss peaks on internal friction spectra $\lambda =f\left(T\right)$, an anomalous change in the frequency of free damped oscillations occurs on the dependencies $\nu =f\left(T\right)$, and accordingly, in the shear modulus $G\left(T\right)$. In this case, a significant deviation of the experimental curve from the proportional theoretical temperature dependence $G=f\left(T\right)$ or $\nu =f\left(T\right)$ is observed. To describe this anomaly, the concept of shear modulus defect ΔG or frequency defect Δν is introduced.

To determine the mechanism of internal friction for the dissipative processes discovered on the internal friction spectrum $\lambda =f\left(T\right)$, the calculation of the magnitude and sign of the shear modulus defect was performed based on the temperature dependence of the frequency of the oscillatory process according to the relation [40]:

(15) $$\Delta G\left(T\right)=\frac{G_{0}\left(T_{0}\right)-G_{i}\left(T_{i}\right)}{G_{0}\left(T_{0}\right)}=\frac{\nu_{0}^{2}\left(T_{0}\right)-\nu_{i}^{2}\left(T_{i}\right)}{\nu_{0}^{2}\left(T_{0}\right)}.$$

This calculation was performed for the α-relaxation process. However, due to the fact that the peak is complex and on the temperature–frequency dependence in the temperature range of manifestation of this process, first an increase in frequency is observed (Figure 9b, Figure 12b, Figure 15b and Figure 18b, red oval marked *1*), and then a decrease (Figure 9b, Figure 12b, Figure 15b and Figure 18b, red ovals marked *2*–*4*); the calculation of ΔG was performed for these two ranges, respectively (Table 4).

The shear modulus defect can have a positive value for dissipative processes of a relaxation nature (*2*–*4*-processes) and a negative value for dissipative processes of a non-relaxation nature (*1*-process). The obtained values of the shear modulus defect allow quantitatively determining the real change in the strength characteristics of the studied materials, taking into account the local temperature changes in the shear modulus caused by local dissipative losses contributed by each dissipative process and manifested on the internal friction spectrum $\lambda =f\left(T\right)$.

**Figure 23:**
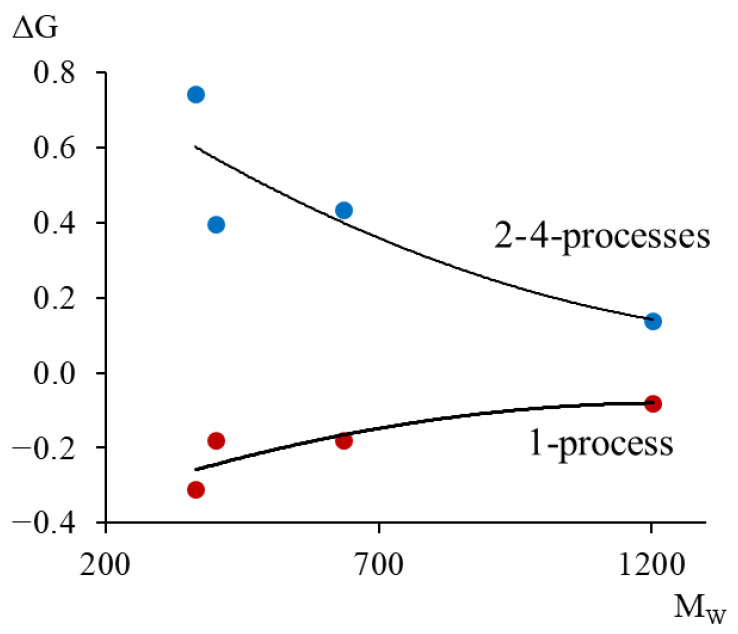
Dependence of the shear modulus defect for γ-, β-, and α-dissipative relaxation processes on the average molecular weight.

From the calculated data (Table 4), graphically presented in Figure 23, we see that with increasing molecular mass, the values of the shear modulus (in absolute value) decrease. This indicates that with increasing molecular mass, the oligomer’s ability to elastically resist external influences increases.

**Figure 24:**
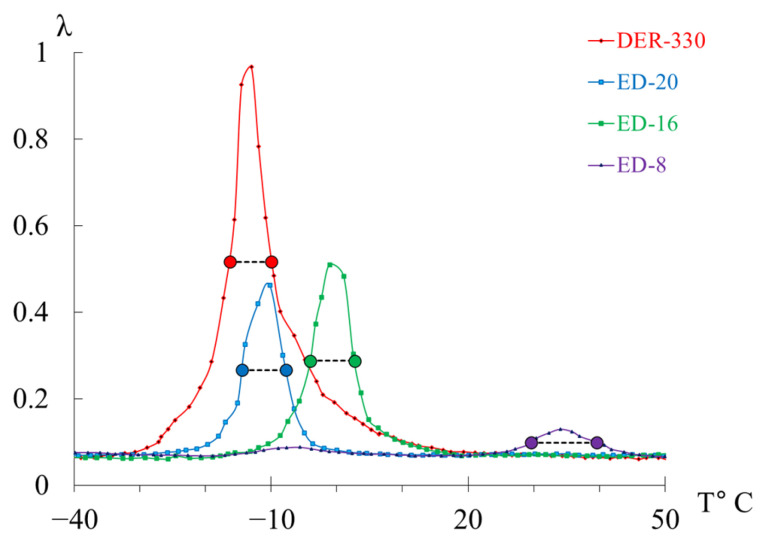
Internal friction spectrum $\lambda =f\left(T\right)$ for epoxy oligomers: DER-330 (red line), ED-20 (blue line), ED-16 (green line), and ED-8 (violet line) in the temperature range from −40 °C to +50 °C.

In Figure 24, the width of the α-relaxation peak at half its height (subtracting the background on which the peak is superimposed) is marked by a black dashed line. At the intersection points of the dashed line and the dissipative loss peak itself, temperatures were determined for the further calculation of relaxation times at these points using Equation (8), where E is the activation energy of the α-relaxation process calculated earlier (Table 3); $\tau_{0}\approx 5\cdot 10^{-12},\;s$ for the α-relaxation process—the theoretical value of the pre-exponential factor.

Thus, we obtained the temperature range at half the height of the peak, which slightly increases with increasing molecular mass of the oligomers, and the range of relaxation time changes Δτ at half the height of the peak (Table 5), which actually characterizes the relaxation micro-heterogeneity of the process (in the first approximation).

The small values of the temperature range and the relaxation time range (Table 5) indicate that the system is relatively homogeneous in terms of the presence of various subsystems. This is clearly seen when comparing the corresponding characteristics of different materials, for example, the relaxation micro-heterogeneity of the epoxy oligomers presented in this work and the relaxation micro-heterogeneity of polyethylene, polyvinyl alcohol, etc. [78].

## 4. Conclusions

1. A deep literature analysis was conducted on studies of the relaxation behavior of epoxy oligomers. An experimental investigation was performed, followed by theoretical analysis of the obtained results for uncured epoxy oligomers, taking into account their aggregation state over a wide temperature range (from −150 °C to +150 °C) in dynamic mode.
2. From the obtained experimental results, it was established that three processes of dissipative losses are detected on the internal friction spectra: *γ*-, *β*-, *α*-, and the *l*-*l* region. The structural rationale for the manifestation of each of these local dissipative processes is considered as follows: the *γ*-process–oscillatory-rotational movements of side groups; the *β*-process–oscillatory-rotational movements of atoms and atomic groups around the axis of the main polymer chain; the *α*-process is associated with the defrosting of the mobility of macromolecular segments and corresponds to mechanical and structural glass transition processes. These processes, in turn, represent a set of dissipative processes superimposed on each other, which manifests itself in the splitting of these loss peaks into components.
3. Mathematical processing of the temperature dependence of the frequency of the free damped oscillatory process made it possible to assume mechanisms of internal friction (relaxation and phase) and to calculate the defect of the shear modulus for the main *α*-relaxation process. It was established that with increasing molecular mass, the ability of the uncured oligomer to elastically resist external shear influences increases in the temperature range of the manifestation of the *α*-relaxation process.
4. The physicochemical characteristics (relaxation time and activation energy) of the γ-, β-, α-local dissipative processes were calculated. The obtained activation energy values are as follows: γ-process–~35–40 kJ/mol; β-process–~40–60 kJ/mol; α-process–~50–60 kJ/mol. The greatest changes in the values of temperature and activation energy (increase in values) with increasing molecular mass of the oligomer, decreasing proportion of epoxy groups, are observed in the region of the α-relaxation process manifestation: temperature increases from −13 °C to +34 °C, activation energy increases from 50 kJ/mol to 60 kJ/mol.
5. It was established that the higher values of the activation energy of the *γ*-relaxation process (compared to the literature data) are associated not only with the temperature position of this process, but also with the frequency value and, to a greater extent, with the value of the pre-exponential coefficient in the Arrhenius equation.
6. An assumption is given that, from the point of view of structural structure, higher values of the activation energy of the *γ*-relaxation process may be associated with the complication of the oligomer system’s structure and a change in the number of intermolecular interactions due to the presence of mono- and non-functional molecules.
7. The possibility of describing the relaxation microinhomogeneity of the *α*-relaxation process depending on the molecular mass of the oligomer is considered.

Thus, the obtained results help us to understand the structure and properties of epoxy oligomers before curing, optimize storage conditions and processing, and provide a preliminary forecast of the material’s behavior during curing and in the cured state.

## Figures and Tables

**Table 1:** Properties of uncured epoxy oligomers [118].

Characteristic	DER-330	ED-20		ED-16		ED-8	
Grade	–	Extra Class	Class I	Extra Class	Class I	Extra Class	Class I
Appearance	Viscous transparent liquid	Viscous transparent liquid		Highly viscous transparent liquid		Solid transparent oligomer	
Color (Co–Fe scale, max)	–	3	8	3(4)	8	2	6
Density at 25 °C, g/cm^3^	1.13	1.166	1.15	1.160	1.160	–	–
Dynamic viscosity at 25 °C, Pa·s	7–10	13–20	12–25	5–18 (50 °C)	5–20 (50 °C)	–	–
Dynamic viscosity at 50 °C, Pa·s	–	5	18	5	20	–	–
Weight-average molecular weight	364	403		635		1203	
Number of fractions	2	3		8		13	
Epoxy equivalent, g/mol	176–185	195–216	195–216	239–269	239–269	430–506	430–537
Mass fraction of epoxy groups, %	23.2–24.4	20–22.5	20–22.5	16–18	16–18	8.5–10	8–10
Chlorine ion content, %, ≤	0.005	0.001	0.005	0.002	0.004	0.001	0.003
Washable chlorine, %, ≤	0.5	0.3	0.8	0.3	0.5	0.2	0.3
Hydroxyl group content, %, ≤	–	17	–	25	–	–	–
Volatile content, %, ≤	0.7	0.2	0.8	0.2	0.4	0.2	0.3
Gelation onset at 30 °C, min	57	24		15 [119]		–	
Activation energy E, kJ/mol	91	102		144		135 [120]	

**Table 2:** Molecular characteristics of commercial diane epoxy oligomers [122,124].

*n*	$\mathrm{Average}\;\mathrm{Molecular}\;\mathrm{Weight}\;\mathrm{of}\;\mathrm{the}\;\mathrm{Fraction},\;M_{W}$	Mass Fraction of Epoxy Oligomer Fraction			
		DER-330	ED-20	ED-16	ED-8
0	340	0.92	0.81	0.425	0.14
1	624	0.076	0.162	0.30	0.176
2	908	0.004	0.0243	0.15	0.165
3	1192	-	0.0032	0.072	0.14
4	1476	-	0.0004	0.031	0.11
5	1760	-	-	0.013	0.08
6	2044	-	-	0.005	0.06
7	2328	-	-	0.002	0.04
≥8	-	-	-	0.002	0.089
Average value		364	403	635	1203

**Table 3:** The main physicomechanical and physicochemical characteristics for all processes of dissipative losses.

Grade	T_max_, (K)	T_max_, (°C)	$\lambda_{max}$	$\nu_{max},\;Hz$	*U*, kJ/mol	$\tau_{max},s$
γ-process						
DER-330	160	−113	0.052	2.62	35.5	0.061
ED-20	157	−116	0.053	2.24	35.0	0.071
ED-16	158	−115	0.056	2.26	35.2	0.070
ED-8	167	−106	0.056	1.90	37.6	0.084
β-process						
DER-330	192	−81	0.069	2.46	42.6	0.065
ED-20	192	−81	0.075	2.12	42.8	0.075
ED-16	193	−80	0.083	2.14	43.1	0.074
ED-8	220	−53	0.080	1.78	49.4	0.090
	267	−6	0.088	1.66	60.3	0.096
α-process						
DER-330	260	−13	0.966	2.31	50.5	0.069
ED-20	263	−10	0.462	1.94	51.4	0.082
ED-16	272	−1	0.510	2.05	53.1	0.078
ED-8	307	34	0.129	1.62	60.5	0.098

**Table 4:** Experimental frequency values, as well as calculated values of shear modulus defects for the *1*–*4* processes of the α-peak of dissipative losses.

Grade	Frequency Range, Hz		Shear Modulus Defect ΔG
*1*-process			
DER-330	2.763	3.165	−0.312
ED-20	2.074	2.253	−0.180
ED-16	2.172	2.358	−0.178
ED-8	1.613	1.676	−0.079
*2*–*4*-process			
DER-330	3.165	1.602	0.744
ED-20	2.253	1.749	0.397
ED-16	2.358	1.774	0.434
ED-8	1.676	1.556	0.138

**Table 5:** Experimental and calculated values of temperatures, relaxation times and their ranges for the *1*–*4* processes of the α-peak of dissipative losses.

Grade	T_1_, °C	T_2_, °C	Δ T, °C	τ_1_, Hz	τ_2_, Hz	Δτ, Hz
DER-330	−16	−10	6	0.093	0.053	0.040
ED-20	−14	−8	7	0.121	0.066	0.055
ED-16	−4	3	7	0.100	0.056	0.044
ED-8	30	40	10	0.139	0.064	0.075

## Data Availability

Data are available upon request to the corresponding author.
